# Hearing-Functioning Problems Across High-Burden Adult Health Conditions in Africa

**DOI:** 10.3390/ijerph23080974

**Published:** 2026-07-28

**Authors:** Gouwa Dawood, Rentia Maart, Quinette Abegail Louw

**Affiliations:** 1 Division of Speech-Language and Hearing Therapy, Department of Health and Rehabilitation Sciences, Faculty of Medicine and Health Sciences, Stellenbosch University, Cape Town 7505, South Africa; 2 Department of Health and Rehabilitation Sciences, Faculty of Medicine and Health Sciences, Stellenbosch University, Cape Town 7505, South Africa; 14572044@sun.ac.za (R.M.); qalouw@sun.ac.za (Q.A.L.)

**Keywords:** hearing-functioning problems, rehabilitation, vestibular dysfunction, Africa, hearing loss, International Classification of Functioning, Disability and Health (ICF)

## Abstract

**Highlights:**

**Public health relevance—How does this work relate to a public health issue?**

- Hearing-functioning problems in African populations frequently extend beyond hearing loss alone and involve overlapping auditory, vestibular, and sensory dysfunction.
- Human immunodeficiency virus/acquired immunodeficiency syndrome (HIV/AIDS), tuberculosis (TB), diabetes mellitus (DM), and headache-related conditions were commonly associated with multidimensional hearing-functioning problems across African settings.

**Public health significance—Why is this work of significance to public health?**

- Current hearing-health research and services in Africa remain largely focused on impairment-level hearing loss, potentially overlooking broader rehabilitation needs.
- This study highlights substantial regional, contextual, and healthcare-system variability in reported hearing-functioning problems across Africa.

**Public health implications—What are the key implications or messages for practitioners, policy makers and/or researchers in public health?**

- Rehabilitation-oriented hearing assessment approaches should consider vestibular, balance, and auditory-processing difficulties alongside hearing loss.
- Greater investment is needed in multidisciplinary hearing and rehabilitation services, particularly within community, rural, and under-resourced healthcare settings.

**Abstract:**

Hearing-functioning problems contribute substantially to disability and rehabilitation needs globally; however, evidence describing the range and interconnected nature of these problems across African populations remains fragmented. This study aimed to describe hearing-functioning problems associated with high-burden adult health conditions across African populations using data from the Rehab4All database. A secondary exploratory analysis was conducted using data extracted from a large Africa-wide scoping review of functioning problems associated with conditions contributing substantially to years lived with disability (YLD). The parent review systematically searched PubMed, Scopus, Web of Science, EBSCOhost, and SABINET and included English-language studies conducted in African adults. Hearing-functioning problems were classified according to the International Classification of Functioning, Disability and Health (ICF). Descriptive analyses, visual mapping techniques, and exploratory random-effects prevalence meta-analyses were performed. Ninety-seven articles published between 2007 and 2022 were included. Most studies originated from Southern Africa, particularly South Africa. Human immunodeficiency virus/acquired immunodeficiency syndrome (HIV/AIDS), tuberculosis (TB), diabetes mellitus (DM), hearing-loss cohorts, and headache-related conditions contributed most frequently to reported hearing-functioning problems. Hearing loss, tinnitus, and vestibular dysfunction were the most commonly reported problems; however, multidimensional co-occurring auditory and vestibular profiles were frequently identified. Distinct functional patterns emerged across conditions, with TB demonstrating predominantly auditory dysfunction, while HIV/AIDS and DM demonstrated broader auditory–vestibular involvement. Hearing-functioning problems within the African evidence base appear multidimensional, interconnected, and unevenly distributed geographically.

## 1. Introduction

Hearing loss is a major public health issue in Africa, affecting millions of people and placing a significant economic burden on society. Currently, approximately 40 million people in Africa experience hearing loss, representing a prevalence rate of 3.6% [1]. Projections indicate a marked increase, with the number of individuals with disabling hearing loss expected to reach 54 million by 2030 and 97 million by 2050 [1]. These trends highlight the growing need for effective and sustainable responses to hearing loss across the region.

The economic implications of untreated hearing loss are equally alarming. Failure to address hearing loss is estimated to cost African economies approximately US$ 27.1 billion annually, driven by reduced productivity, increased healthcare expenditure, and reduced quality of life for individuals with hearing impairment [1].

Despite this substantial and growing burden, the evidence base on hearing-related functioning problems among adults in Africa remains limited. Available studies often rely on non-representative populations, such as older adults or individuals with condition-specific hearing loss. A recent Africa-wide systematic review identified only seven eligible studies (*n* = 2761), with most research conducted among specific populations such as those of retirement homes and of schools for students with special educational needs, and with a predominance of female participants, limiting generalisability [2].

When framed within the International Classification of Functioning, Disability and Health (ICF), the existing literature suggests that while impairment at the level of body functions is well documented, the broader disability burden associated with hearing is mediated through activity limitations and participation restrictions, shaped by environmental and contextual factors rather than hearing thresholds alone [3]. Where more detailed evidence is available, hearing loss is consistently associated with reduced social participation, poorer emotional well-being, and adverse cognitive and psychosocial outcomes among older adults [2,4,5,6]. In addition, evidence from sub-Saharan Africa highlights persistent barriers to healthcare access for individuals with hearing impairment, including communication difficulties, discrimination, financial constraints, and negative healthcare-worker attitudes, as well as limited autonomy, security, and privacy [7].

Considering the high prevalence of hearing loss and its sequelae, and the lack of evidence synthesis of the prevalence of hearing problems in Africa, this scoping review aimed to map and synthesise evidence on hearing-related functioning problems among adults in Africa using data from the Rehab4All database [8]. Specifically, the study sought to describe the types and prevalence of hearing-related functioning problems reported as sequelae of the top health conditions contributing to the most years lived with disability (YLD), identify the conditions most frequently associated with these problems, map their distribution across countries, and examine the levels of care at which they are reported.

## 2. Materials and Methods

### 2.1. Study Design

This study presents a secondary exploratory analysis of hearing-functioning problems extracted from a large scoping review that mapped functioning problems associated with major disability-causing health conditions among adults in all African countries. Data were extracted using the Rehab4All application (https://ttlx.me/rehab4all, accessed on 21 July 2026), a bespoke secure web-based application developed to support structured data extraction and classification of functioning problems using the ICF [9]. The parent scoping review followed the Arksey and O’Malley framework [10], the updated Joanna Briggs Institute (JBI) methodology for scoping reviews [11] and the PRISMA-ScR reporting guidelines. As is typical of scoping reviews, the underlying evidence comprised studies with diverse methodological designs, reflecting the breadth of the available literature rather than a homogeneous body of evidence.

### 2.2. Parent Scoping Review and Data Source

The methods of the parent scoping review, including the search strategy, eligibility criteria, study selection process, and data extraction procedures, have been published elsewhere [12]. The broader review included peer-reviewed studies from African countries published from January 2006 onwards which were identified through searches of PubMed, Scopus, Web of Science, EBSCOhost, and SABINET [12]. Searches for potential articles started as early as August 2021 and updated searches were conducted from August 2024. Due to time and resource constraints, only English-language studies were included, as limited multilingual proficiency of the research team and the absence of professional translation services prevented the accurate extraction and assessment of eligible data. Studies were included if they reported on functioning problems amenable to rehabilitation in African adults (15 years and older) which resulted from at least one diagnosed health condition in the top ten health conditions contributing to highest YLDs per country (based on the Global Burden of Disease [GBD] 2019 estimates obtained from the GBD Compare tool on https://vizhub.healthdata.org/gbd-compare/, accessed on 1 July 2021).

Extracted data from the Rehab4All online web application, included study characteristics, population details, health conditions (classified using the *International Classification of Diseases, 11th Revision* and GBD groupings), functioning problems, outcome measures, prevalence estimates, country, age group, and level of care. Data extraction for the parent scoping review was performed independently by two reviewers with verification by a third reviewer, as described in the published parent review [12].

The present analysis used the final, cleaned extraction database generated by the parent scoping review. No additional searches or data extraction were conducted.

### 2.3. Eligibility Criteria for the Hearing-Focused Analysis

For this secondary analysis, a subset of records was identified from the Rehab4All database based on predefined hearing-specific eligibility criteria. Hearing-functioning problems were identified and classified according to the ICF framework, primarily within the sensory functions domain (b2), including hearing functions (b230) and sensations associated with hearing and vestibular function (b240) [3]. Eligible studies were identified by filtering the Rehab4All database for records coded under the relevant ICF hearing and sensory functioning categories and reviewing extracted functioning descriptions to ensure alignment with the aims of the present analysis.

### 2.4. Data Analysis and Synthesis

The extracted hearing-functioning problems from the Rehab4All application included hearing loss, tinnitus, vestibular impairment, dizziness/vertigo, falls, fear of falling, pain, fear of sound, difficulties understanding others, and unspecified hearing impairment. Conceptually related hearing-functioning problems extracted from the Rehab4All application were grouped into standardised functional domains to facilitate descriptive synthesis and comparison across health conditions. These groupings were informed by the organisation of functioning problems within the Rehab4All application, the ICF framework, and author consensus to facilitate descriptive synthesis and comparison across health conditions. “Hearing Loss” included both hearing loss and unspecified hearing impairment, while “Vestibular Impairment” included dizziness/vertigo and vestibular impairment. Falls and fear of falling were grouped under “Balance-related Problems,” representing functional problems associated with postural stability. Although vestibular impairment and balance-related problems are clinically related, they were analysed as separate categories because vestibular dysfunction does not necessarily result in balance problems, and balance problems may arise from multiple non-vestibular causes. Some functioning problems, such as tinnitus, remained as standalone categories due to their distinct clinical presentation. Broader categories were also created where appropriate, with pain incorporated under “Other Auditory Symptoms,” and difficulties understanding others grouped under “Auditory-processing Difficulties”. Fear of sound was categorised as “Phonophobia” to reflect its distinct clinical presentation. Grouping individual functioning problems into broader clinically meaningful categories allowed comparable synthesis across studies, while preserving distinctions between related but conceptually different impairments.

Hearing-functioning problems were profiled descriptively according to underlying health conditions contributing substantially to YLD in Africa. Descriptive analyses included the frequency of reported hearing-functioning problems, average reported prevalence estimates, total sample sizes, number of included studies, geographic distribution across African regions and countries, and levels of care where functioning problems were identified.

Cross-tabulations were performed to explore relationships between health conditions and hearing-functioning problems. Findings were summarised using descriptive statistics, summary tables, heatmaps, radar plots, network visualisations, and narrative synthesis to illustrate multidimensional hearing-function profiles across conditions.

For hearing-functioning problem categories with sufficient available study-level prevalence data, exploratory prevalence meta-analyses were conducted using random-effects models to generate pooled prevalence estimates and forest plots. These analyses were performed to further explore the distribution and variability of selected hearing-functioning problems within the available African evidence base. Due to anticipated methodological and clinical heterogeneity across included studies, including differences in study design, populations, settings, and outcome measures, pooled prevalence estimates were interpreted cautiously and considered exploratory rather than representative population prevalence estimates.

## 3. Results

**Figure 1:**
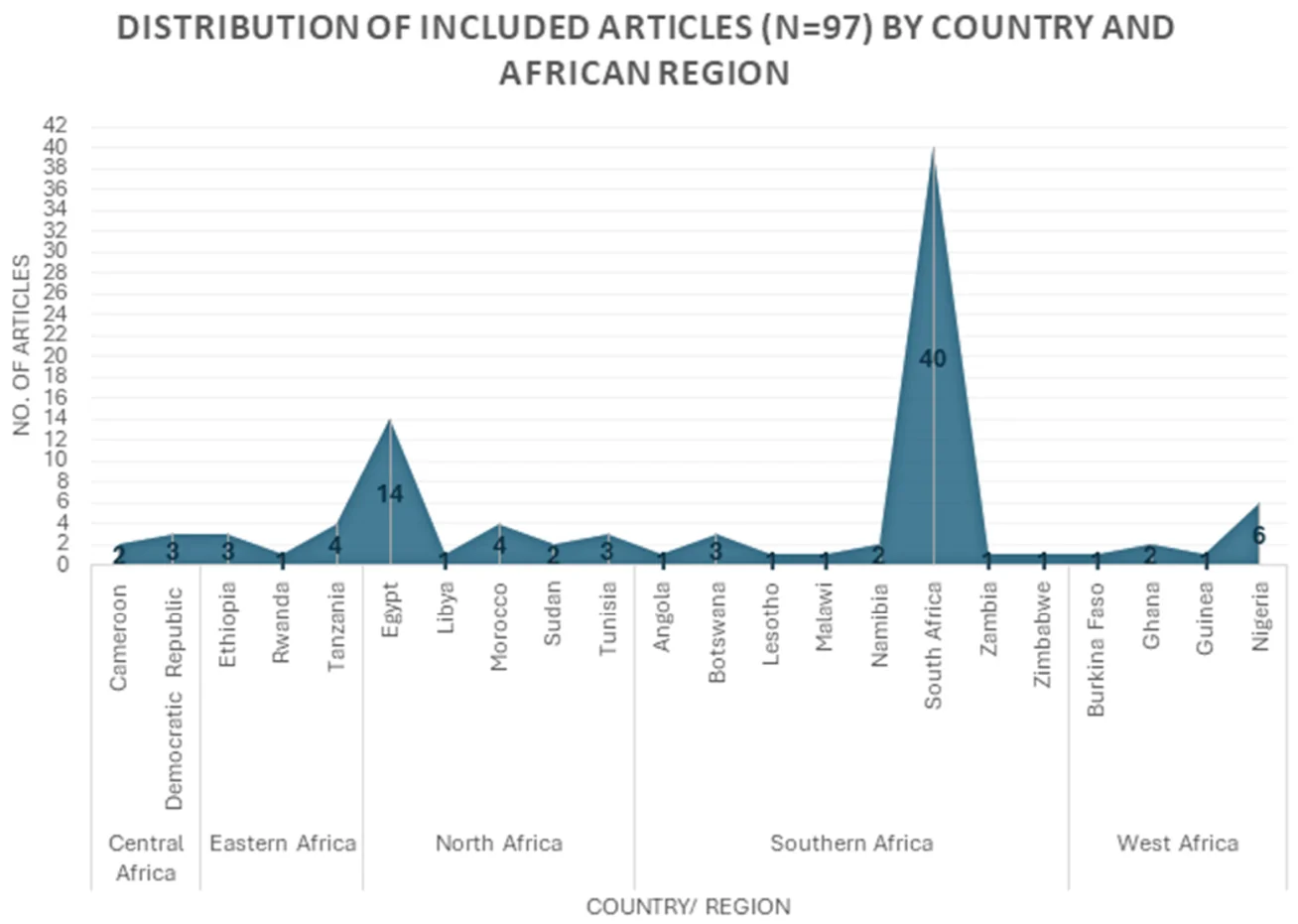
Number of included articles per country/region.

A total of 97 articles reporting on hearing functioning problems in adults from 22 African countries were extracted from the Rehab4All database and included in our analysis. Articles were published between 2007 and 2022 and a combined sample of 14,208 male and female participants over the age of 15 years old suffering from at least one of the top conditions contributing to YLD in Africa were included. The age distribution of participants varied across the included studies according to the underlying health condition, with age reported inconsistently as categories, ranges, means, or medians. Most of the studies were from the Southern African region (*n* = 50, 64%); of these, 40 were from South Africa (Figure 1). Ten articles originated from Western Africa, five from Central Africa and eight from Eastern Africa. Northern Africa yielded 24 articles.

### 3.1. Demographic Details

The included studies represented a range of study designs, with cross-sectional studies (*n* = 33, 34%), cohort studies (*n* = 23, 24%), and case studies/case series (*n* = 22, 23%) being the most used. Together, these designs accounted for approximately 80% of the included studies. Only one study utilised a survey design.

More than one-third of the studies were conducted at hospital level (*n* = 34, 35%), while almost 30% (*n* = 29) were conducted in primary healthcare clinics. Specialised facilities accounted for 14% (*n* = 14) of the included studies, whereas only two studies were conducted within rehabilitation facilities. The level of care was not specified in 10 studies, and 8% of studies (*n* = 8) were conducted within community settings.

In terms of setting, half of the studies were conducted in urban areas, while more than 40% did not report the study setting. Very few studies were conducted in rural (*n* = 5, 5%), semi-rural (*n* = 1, 1%), or mixed rural and urban settings (*n* = 1, 1%). Table 1 below summarises the study designs, levels of care, and study settings of the included studies.

### 3.2. Most Frequently Reported Health Conditions Contributing to Hearing-Functioning Problems

Table 2 presents the different health conditions (*n* = 26) associated with hearing-functioning problems among adults in Africa. To facilitate the analysis, similar health conditions were grouped into broader disease categories (*n* = 12). Because hearing loss was identified both as a health condition and as a hearing-functioning problem within the dataset, a distinction was made between the two during analysis. Studies in which participants were recruited primarily based on hearing loss as a health condition were classified as “hearing loss cohorts,” while hearing loss reported as a functioning problem remained classified under hearing loss.

HIV/AIDS, TB, and hearing loss cohorts contributed the largest proportion of studies within the dataset, with 31, 16, and 20 articles, respectively. Given the high prevalence of HIV/AIDS and TB co-occurrence within the African context, studies reporting on HIV/AIDS only and those reporting on HIV/AIDS with TB co-infection were initially presented as separate categories in Table 2 but were combined under the broader HIV/AIDS category for all subsequent descriptive and exploratory analyses (e.g., Table 3). In addition, DM, headaches, and stroke each contributed seven articles. In contrast, hearing-functioning problems were less frequently reported among individuals with mental health conditions, musculoskeletal conditions, visual impairments, and conflict- or war-affected populations, with only one to two articles identified for each of these groups.

### 3.3. Reported Prevalence of Hearing-Functioning Problems Across Health Conditions Contributing to YLD

In the table below (Table 3), we present the average reported prevalence of hearing-functioning problems identified within the available African evidence base across the health conditions contributing substantially to YLD. For clarity, the term “hearing-loss cohorts” refers to participant groups recruited on the basis of hearing loss as an underlying health condition, whereas “hearing loss” refers to an ICF-classified hearing-functioning problem reported within the included studies. Hearing loss was the most consistently reported functioning problem across conditions, including cancer-related populations (85.5%), DM (37.6%), TB (35.6%), and stroke (29.5%). Tinnitus was also frequently reported, with especially high prevalence among hearing-loss cohorts (73.3%) and moderate prevalence among TB (31.1%), cancer (30.9%), and DM (28.1%) populations. Vestibular dysfunction demonstrated notable prevalence in headache disorders (46.9%), hearing-loss cohorts (37.8%), and musculoskeletal conditions (37.2%), while balance problems were primarily reported in HIV/AIDS-related populations (27%) and DM (21.8%). Additional hearing-functioning problems, including auditory-processing difficulties (90%), other auditory symptoms (88.4%), and phonophobia (74.3%), were reported predominantly within hearing-loss and headache-related cohorts.

### 3.4. Weighted Pooled Prevalence Estimates for Selected Condition-Functioning Problem Combinations

Where sufficient data were available, we generated exploratory meta-analytic forest plots for selected condition-functioning problem combinations. While Table 3 presents average reported prevalences across the included studies, the forest plots provide weighted pooled prevalence estimates based on study-level sample sizes and prevalence data. Forest plots were generated for commonly reported and sufficiently represented hearing-functioning problems, including hearing loss, tinnitus, and vestibular dysfunction among TB and HIV/AIDS populations. These analyses were conducted to further explore the variability, distribution, and interconnectedness of hearing-functioning problems reported within the African evidence base.

#### 3.4.1. HIV/AIDS and Vestibular Impairment

**Figure 2:**
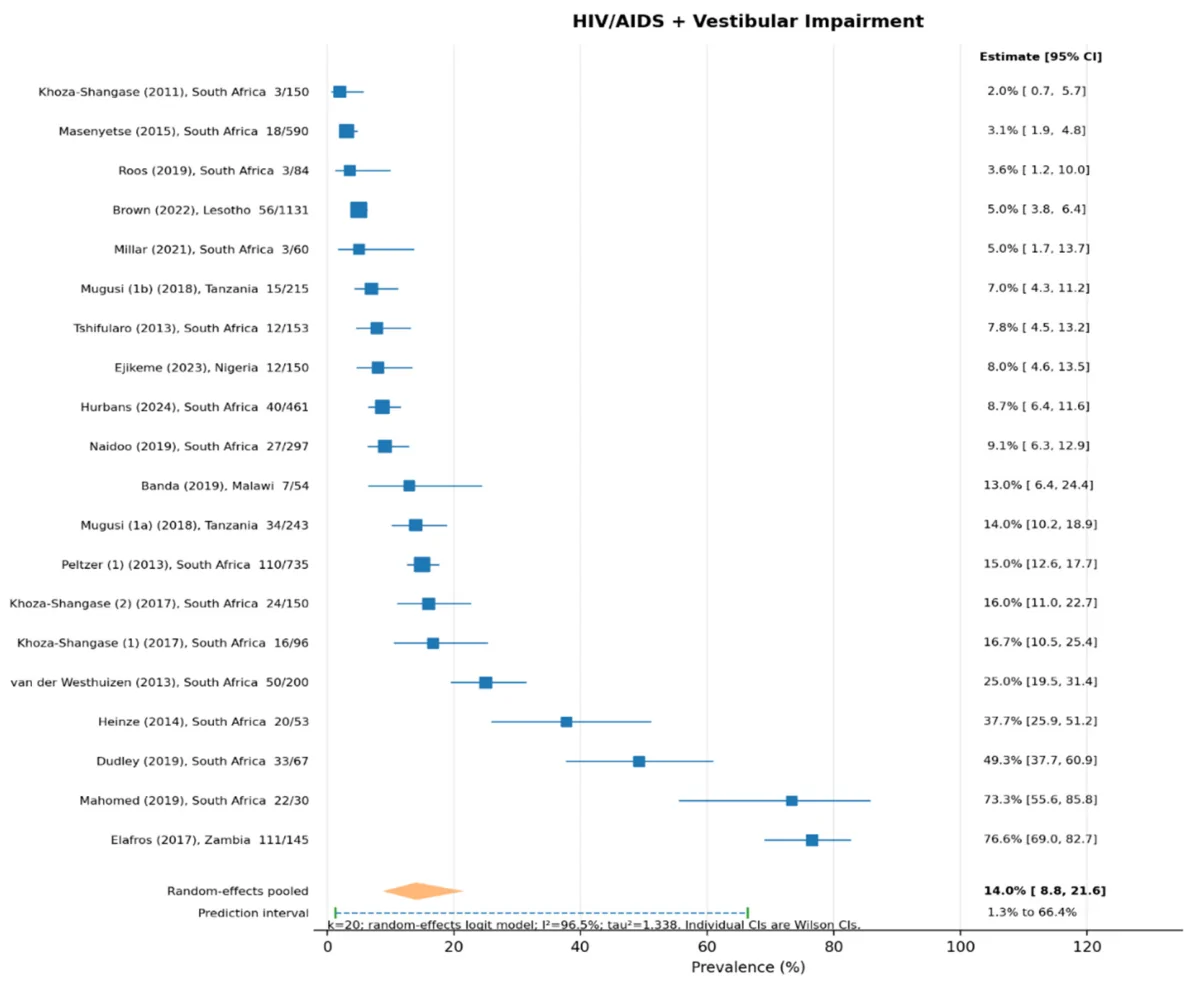
Weighted pooled prevalence estimates of vestibular impairment in individuals with HIV/AIDS across included studies. Study citations are provided within the figure and correspond to the full references listed in the manuscript. Squares represent individual study prevalence estimates (with square size proportional to study weight), horizontal lines indicate 95% confidence intervals, the orange diamond represents the pooled random-effects prevalence estimate, and the dashed line represents the prediction interval. Study citations are provided within the figure and correspond to the full references listed in the manuscript [13,14,15,16,17,18,19,20,21,22,23,24,25,26,27,28,29,30].

**Figure A1:**
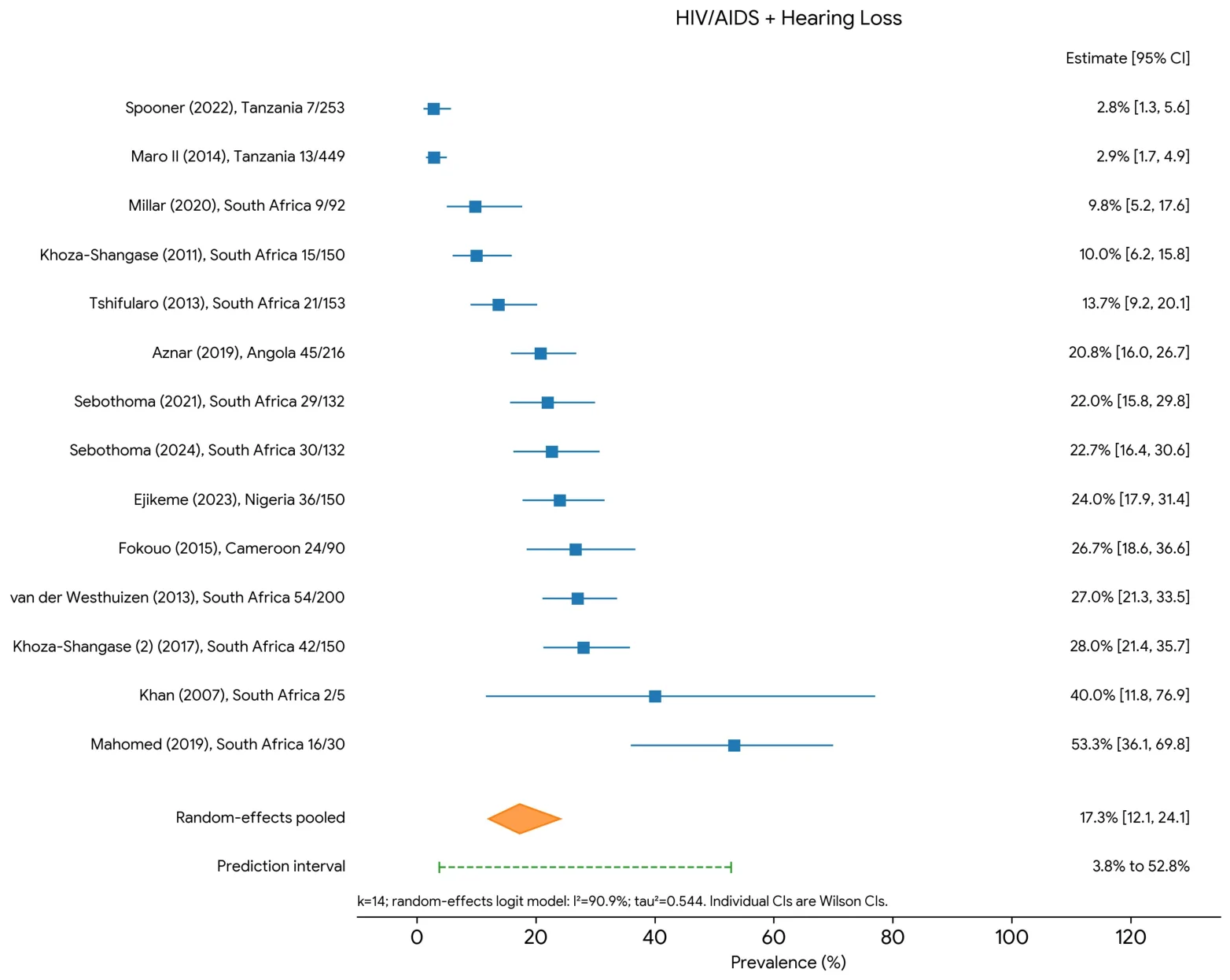
Weighted pooled prevalence estimates of hearing loss in individuals with HIV/AIDS across included studies. Study citations are provided within the figure and correspond to the full references listed in the manuscript. Squares represent the prevalence estimate for each included study, with square size proportional to the study weight. Horizontal lines indicate the 95% confidence intervals. The orange diamond represents the pooled random-effects prevalence estimate, and the dashed line represents the prediction interval [14,15,23,24,28,29,60,61,62,63,64,65,66,67].

**Figure A2:**
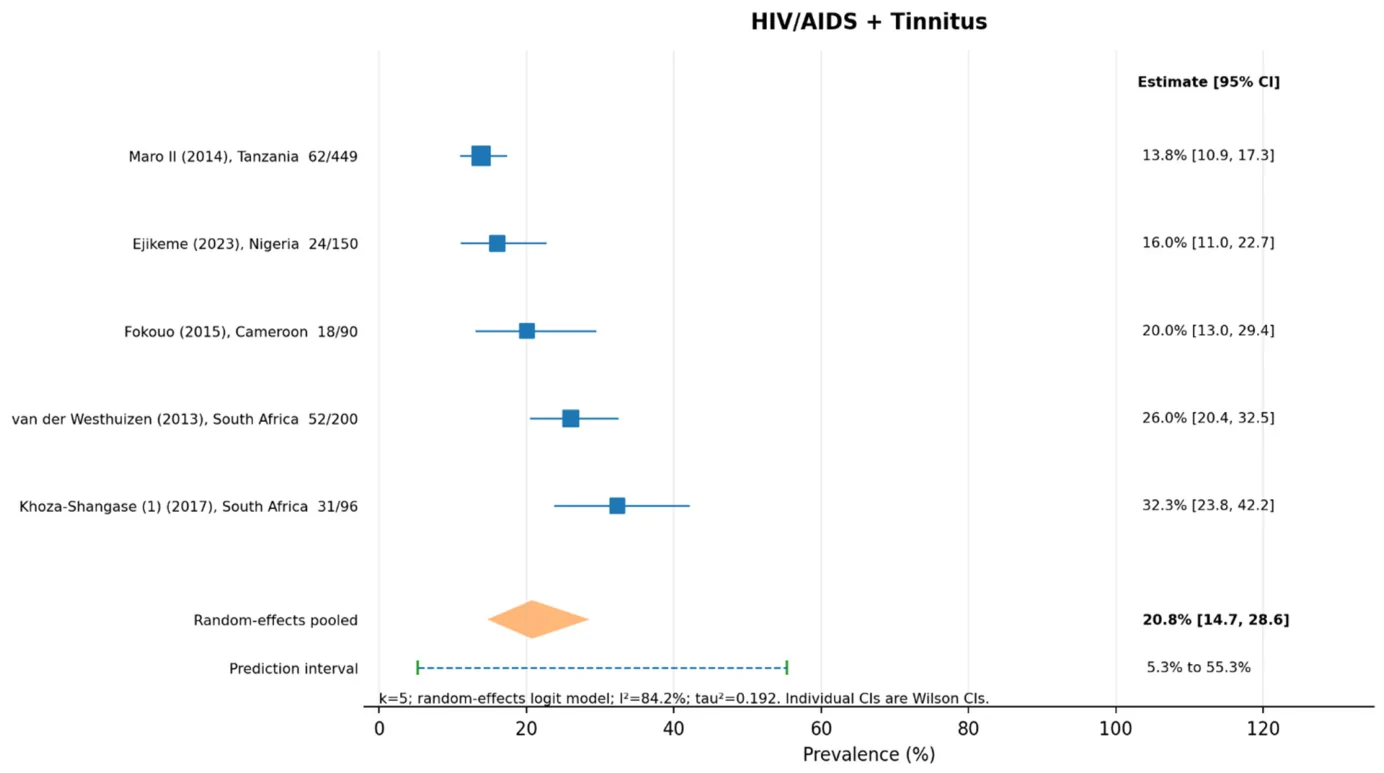
Weighted pooled prevalence estimates of tinnitus in individuals with HIV/AIDS across included studies. Study citations are provided within the figure and correspond to the full references listed in the manuscript. Squares represent the prevalence estimate for each included study, with square size proportional to the study weight. Horizontal lines indicate the 95% confidence intervals. The orange diamond represents the pooled random-effects prevalence estimate, and the dashed line represents the prediction interval [14,24,28,61,62].

An exploratory meta-analytic forest plot of vestibular impairment among adults with HIV/AIDS is presented (Figure 2) to further illustrate the variability and multidimensional nature of hearing-functioning problems across included studies. The forest plot demonstrated substantial variability in reported prevalence estimates across populations and settings, with studies reporting both relatively low and markedly elevated prevalence estimates of vestibular dysfunction. Despite variation in prevalence estimates, vestibular dysfunction was consistently identified across multiple African HIV/AIDS populations. Additional exploratory forest plots for hearing loss and tinnitus are provided in Appendix A (Figure A1 and Figure A2).

#### 3.4.2. TB and Hearing Loss

**Figure 3:**
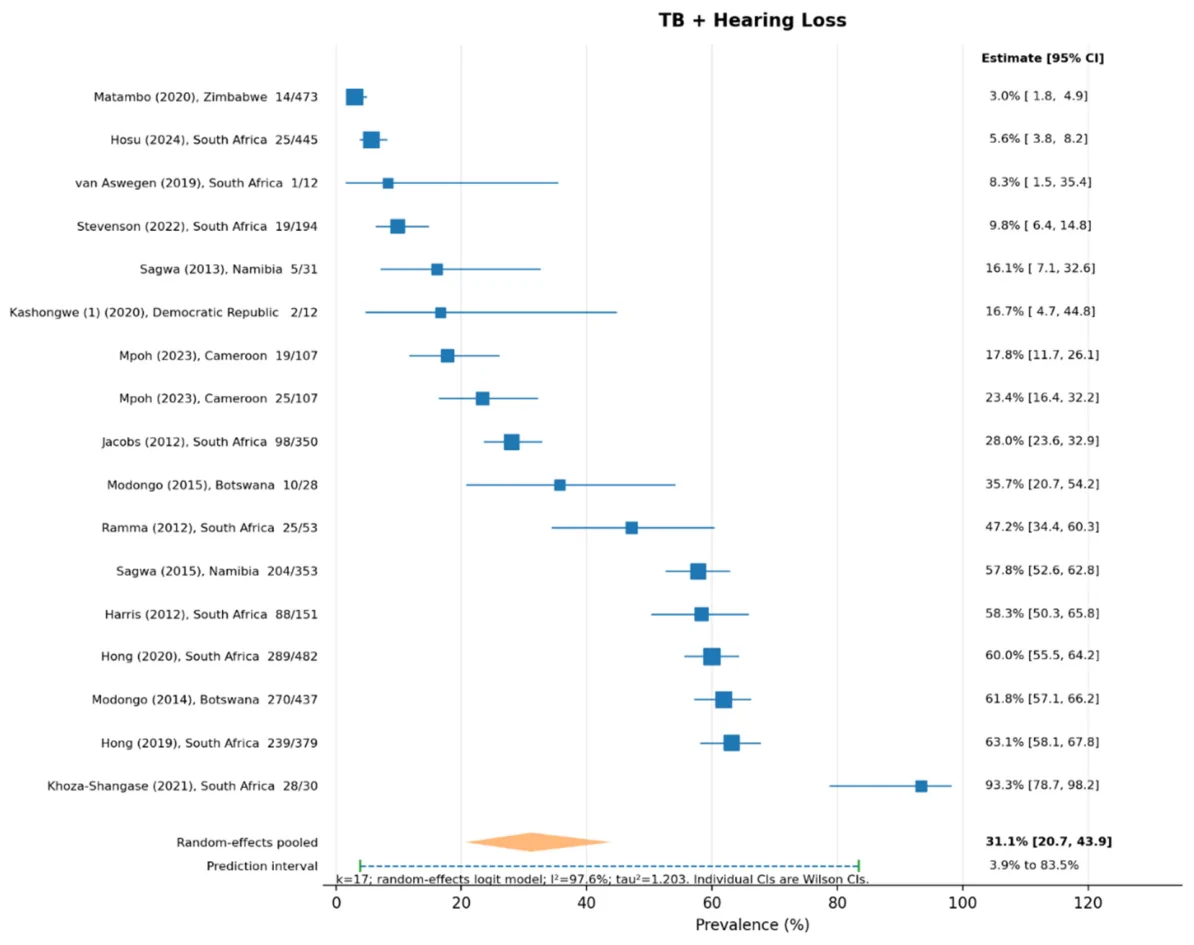
Weighted pooled prevalence estimates of hearing loss among individuals with TB across included studies. Study citations are provided within the figure and correspond to the full references listed in the manuscript. Squares represent individual study prevalence estimates (with square size proportional to study weight), horizontal lines indicate 95% confidence intervals, the orange diamond represents the pooled random-effects prevalence estimate, and the dashed line represents the prediction interval. Study citations are provided within the figure and correspond to the full references listed in the manuscript [31,32,33,34,35,36,37,38,39,40,41,42,43,44,45,46].

**Figure A3:**
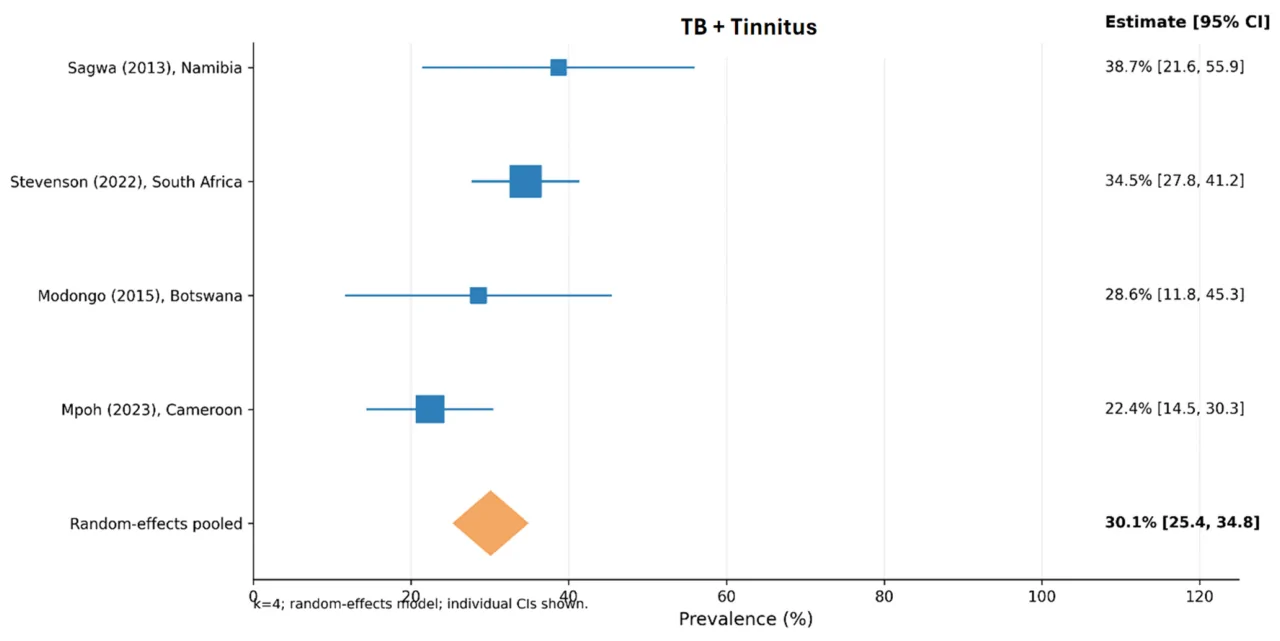
Weighted pooled prevalence estimates of tinnitus in individuals with TB across included studies. Study citations are provided within the figure and correspond to the full references listed in the manuscript. Squares represent the prevalence estimate for each included study, with square size proportional to the study weight. Horizontal lines indicate the 95% confidence intervals. The orange diamond represents the pooled random-effects prevalence estimate, and the dashed line represents the prediction interval [31,33,34,42].

The exploratory forest plot below (Figure 3) illustrates the weighted pooled prevalence estimates of hearing loss among individuals with TB. Compared to the broader multidimensional auditory–vestibular profiles observed in HIV/AIDS populations, TB-related populations demonstrated predominantly auditory dysfunction profiles characterised mainly by hearing loss and tinnitus. Although substantial variability in prevalence estimates was observed across included studies, hearing loss was consistently identified across TB populations and healthcare settings. The recurring predominance of auditory symptoms across the included evidence further supports the interpretation that TB-related hearing-functioning problems within African populations may be strongly influenced by ototoxic treatment exposure and auditory system involvement. Vestibular dysfunction was identified in some TB populations, but appeared comparatively less prominent within the available evidence base. An additional exploratory forest plot for tinnitus in individuals with TB is provided in Appendix A (Figure A3).

### 3.5. Interconnected Hearing-Functioning Profiles Across Health Conditions

**Figure 4:**
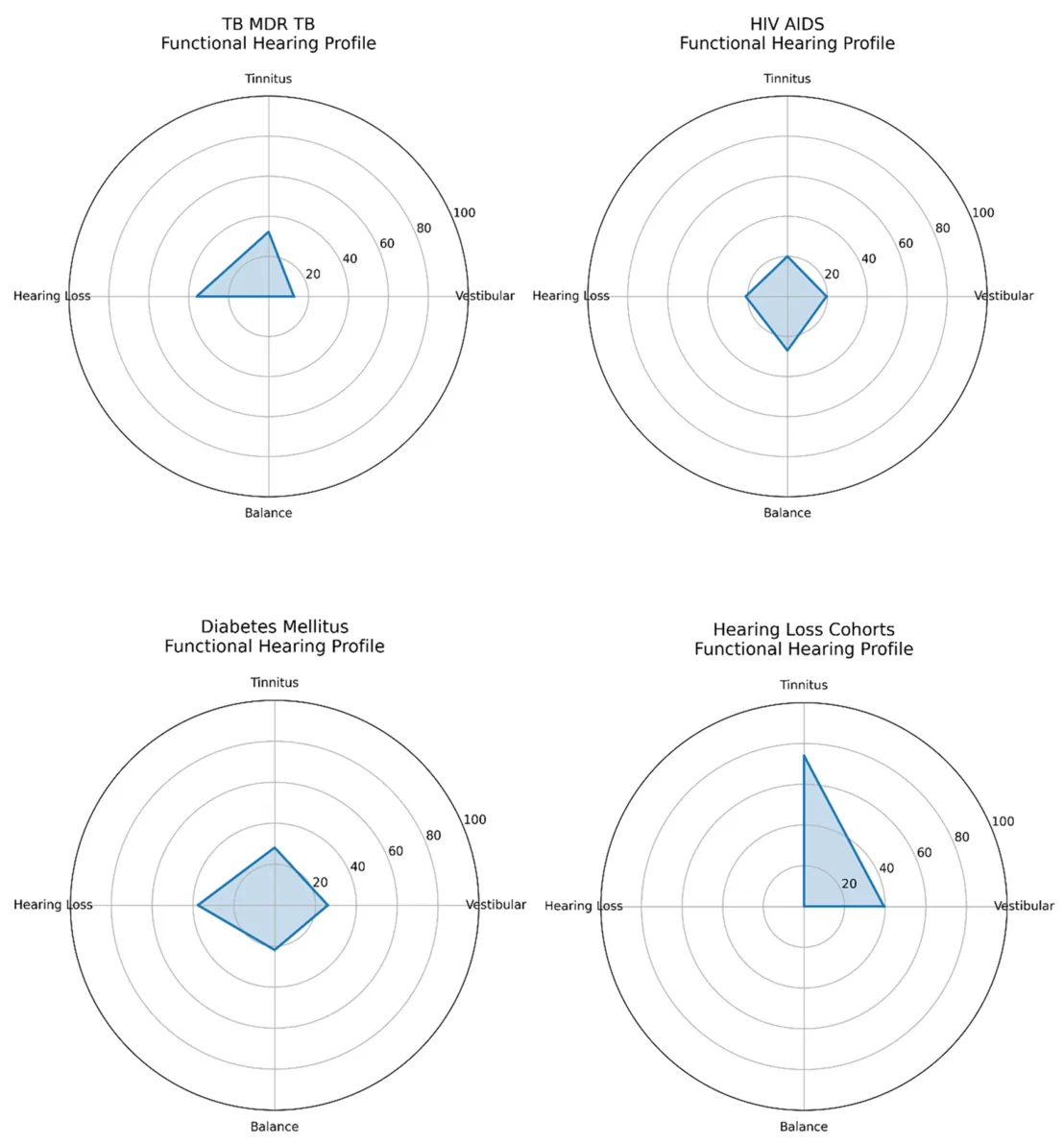
Radar plots illustrating the multidimensional hearing-function profiles associated with TB, HIV/AIDS, DM and Hearing Loss cohorts.

**Figure A4:**
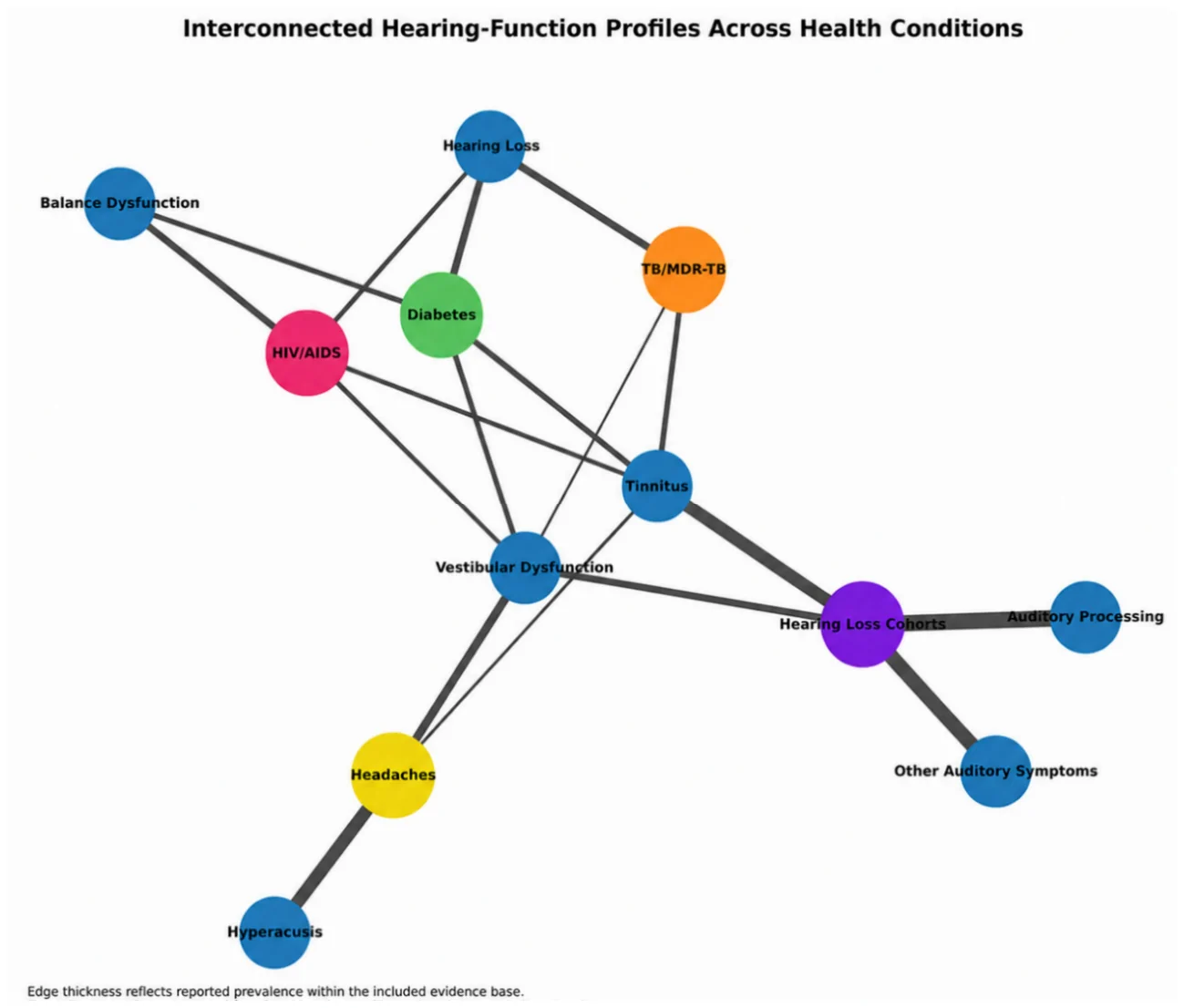
Network illustrating the interconnected hearing-function profiles across health conditions. Blue nodes represent functioning problems, whereas coloured nodes represent the health conditions included in the review. Edge thickness reflects the reported prevalence within the included evidence base.

Our findings indicate that hearing-functioning problems rarely occurred in isolation, with several conditions demonstrating overlapping auditory, vestibular, sensory, and balance-related dysfunction profiles. The radar plots (Figure 4) illustrate distinct multidimensional hearing-function patterns within specific conditions. Furthermore, a network graph (Appendix A, Figure A4) further demonstrates the broader interconnectedness of hearing-functioning problems across conditions that contribute substantially to YLD within the included African evidence base. Tinnitus, vestibular dysfunction, and hearing loss emerged as the most interconnected functioning domains, linking multiple conditions simultaneously.

The radar plots visually demonstrate the relative prominence and co-occurrence of different hearing-functioning problems, including hearing loss, tinnitus, vestibular dysfunction, and balance difficulties, across selected disease categories. TB demonstrated a predominantly auditory profile characterised mainly by hearing loss and tinnitus, with comparatively lower vestibular involvement. In contrast, HIV/AIDS demonstrated a broader multidomain auditory–vestibular profile involving hearing loss, tinnitus, vestibular dysfunction, and balance problems occurring concurrently. Diabetes mellitus also demonstrated a multidimensional profile with relatively balanced representation across hearing loss, tinnitus, vestibular dysfunction, and balance dysfunction. Hearing-loss cohorts demonstrated particularly strong co-occurrence between tinnitus and vestibular dysfunction.

### 3.6. Distribution of Reported Hearing-Functioning Problems Across Africa

**Figure 5:**
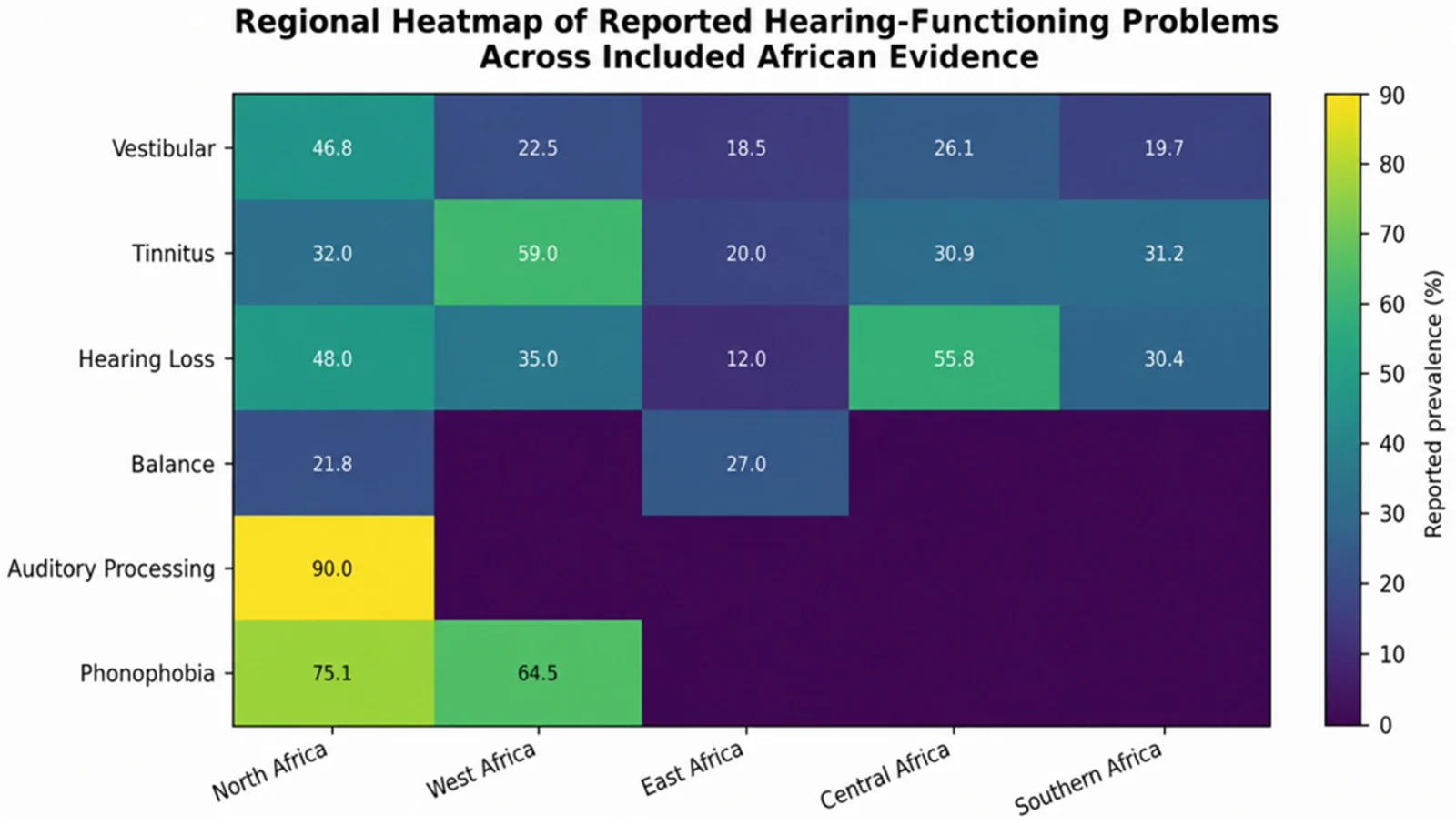
Regional heatmap illustrating the distribution of reported hearing-functioning problems across Africa.

Figure 5 presents a regional heatmap illustrating the distribution of reported hearing-functioning problems across the African evidence base. The heatmap visualises patterns of hearing-function impairment reported within different African regions, including vestibular dysfunction, tinnitus, hearing loss, balance dysfunction, auditory-processing difficulties, and phonophobia. Darker shading represents relatively higher reported prevalence values within the studies included.

Vestibular dysfunction, tinnitus, and hearing loss emerged as commonly reported problems across several regions, while balance dysfunction, auditory processing and phonophobia were less frequently reported across Africa, and not at all in some parts of Africa.

North African studies demonstrated the broadest range of reported functioning domains, including vestibular dysfunction, tinnitus, hearing loss, balance dysfunction, auditory-processing difficulties, and phonophobia. West African evidence showed notable reporting of tinnitus, hearing loss, and vestibular dysfunction, while Central African studies showed higher reported hearing loss. Southern African evidence, although it contributed the largest number of included studies overall, was represented in this heatmap mainly by vestibular dysfunction, tinnitus, and hearing loss.

## 4. Discussion

This scoping review mapped and synthesised evidence on hearing-functioning problems among adults living with conditions contributing substantially to YLD in Africa using data extracted from the Rehab4All database. Overall, the findings suggest that hearing-functioning problems within the African evidence base are multidimensional, interconnected, and contextually variable rather than isolated auditory impairments. HIV/AIDS, TB, DM, Hearing Loss cohorts, and Headache-related conditions contributed most frequently to the range of reported hearing-functioning problems, with several conditions demonstrating overlapping auditory, vestibular, balance-related, and sensory dysfunction profiles. In addition, most of the studies concentrated on the Southern African region. Importantly, this study represents a mapping of the current published African evidence base rather than the true epidemiology or distribution of hearing-functioning problems across the continent. Consequently, the observed patterns should be interpreted as reflecting where and how hearing-functioning problems have been investigated and reported, while simultaneously highlighting important evidence gaps requiring future research.

### 4.1. Co-Occurrence of Functioning Problems

A key finding emerging from this review was the consistent co-occurrence of hearing loss, tinnitus, vestibular dysfunction, auditory-processing difficulties, and phonophobia across several conditions contributing substantially to YLD in Africa. Rather than occurring as isolated impairments, many hearing-functioning problems appeared to cluster together, suggesting broader multidimensional auditory and vestibular involvement across affected populations. Similar patterns have been reported internationally, where hearing loss, tinnitus, dizziness, and vestibular symptoms frequently co-occur across adult populations and are increasingly recognised as interconnected rather than independent clinical entities [47,48].

Distinct functional profiles nevertheless emerged across conditions in our study. TB-related populations demonstrated predominantly auditory dysfunction profiles characterised mainly by hearing loss and tinnitus, likely reflecting the well-established ototoxic effects associated with aminoglycoside exposure in MDR-TB treatment regimens [49,50]. In contrast, HIV/AIDS and DM demonstrated broader auditory–vestibular involvement, including hearing loss, tinnitus, vestibular dysfunction, and balance difficulties occurring concurrently. These broader auditory–vestibular profiles may reflect the multisystem nature of these conditions and the combined effects of chronic inflammation, neuropathic change, vascular compromise, medication exposure, and opportunistic infections [51,52]. Headache-related conditions demonstrated particularly strong vestibular and sound-sensitivity profiles, consistent with recognised features of vestibular migraine and central sensory processing dysfunction [53]. Hearing-loss cohorts further demonstrated substantial overlap between tinnitus, vestibular dysfunction, auditory-processing difficulties, and other auditory symptoms, reinforcing the notion that hearing-related disability frequently extends beyond peripheral hearing impairment alone.

Collectively, these findings suggest that hearing-functioning problems associated with high-burden health conditions in Africa are more complex and multidimensional than is often reflected in routine hearing assessments, highlighting the need for broader rehabilitation-oriented and multidisciplinary models of care. Although the included health conditions differ in their underlying pathophysiology, the aim of this study was to identify shared hearing-functioning profiles from a rehabilitation perspective rather than compare disease-specific mechanisms. This approach reflects the organisation of rehabilitation around functioning rather than diagnosis and may better inform integrated rehabilitation planning across diverse health conditions.

### 4.2. Implications of Vestibular Dysfunction

Vestibular dysfunction emerged prominently across several conditions, particularly among headache-related conditions, hearing-loss cohorts, HIV/AIDS, and DM populations. Despite this, vestibular impairments remain comparatively under-represented within African hearing-health and rehabilitation literature, where clinical assessment has historically focused predominantly on impairment-level hearing loss identified through audiometric thresholds [54,55]. This pattern was reflected within the review itself, where hearing loss remained the most consistently reported functioning problem across conditions. Vestibular dysfunction may have important implications for balance, mobility, falls risk, participation, and quality of life, particularly among older adults and individuals living with chronic conditions [56,57]. Similarly, the identification of auditory-processing difficulties and phonophobia highlights broader sensory and perceptual dimensions of hearing-function impairment that remain insufficiently explored within African rehabilitation research.

### 4.3. Southern Africa

Most included studies originated from Southern Africa, with South Africa contributing the largest proportion of articles within the evidence base. This geographical concentration likely reflects differences in research capacity and publication output rather than the underlying distribution of hearing-functioning problems across Africa. A substantial proportion of these studies focused on hearing-functioning problems associated with HIV/AIDS and multidrug-resistant tuberculosis (MDR-TB), likely reflecting both the high regional burden of infectious diseases and South Africa’s comparatively stronger research infrastructure and scientific output [58]. The predominance of HIV/AIDS- and TB-related hearing-functioning problems within the evidence base further reflects the established association between these conditions, ototoxic medication exposure, and auditory–vestibular dysfunction [49]. While the concentration of evidence around infectious diseases is understandable given the regional disease burden, it may also contribute to the relative under-representation of hearing-functioning problems associated with non-communicable conditions and broader rehabilitation populations.

### 4.4. Geographical Distribution and Level of Care

Regional and service-level variation across the heatmaps suggested that hearing-functioning problems may be investigated, identified, and reported differently across African contexts and healthcare settings. More than half of the included studies originated from South Africa, limiting the representativeness of the evidence across other African regions, particularly West and North Africa. Although Southern Africa contributed the largest proportion of studies overall, North African studies demonstrated the widest range of reported hearing-functioning domains. These differences may reflect variation in research priorities, specialist service availability, clinical assessment approaches, and the extent to which broader vestibular and sensory symptoms are routinely evaluated. Similarly, most evidence originated from hospital- and primary care-level studies conducted within relatively well-resourced urban settings, while rehabilitation facilities, community settings, and rural or semi-rural populations remained comparatively under-represented. This pattern aligns with broader systemic challenges documented across the World Health Organisation (WHO) African Region, including limited workforce capacity, uneven resource distribution, and the concentration of specialised hearing-health services within urban and tertiary-level facilities [1].

These findings therefore represent the distribution of the current evidence base rather than the availability of rehabilitation services or the true burden of hearing-functioning problems across different healthcare settings. Consequently, opportunities for early identification, multidisciplinary assessment, and continuity of rehabilitation-focused care may remain restricted for many affected populations.

### 4.5. Variability in Prevalence Estimates

The exploratory meta-analytic forest plots demonstrated substantial variability in prevalence estimates across studies, particularly among HIV/AIDS- and TB-related populations. This heterogeneity was anticipated given the diversity of included study populations, clinical settings, disease severity, treatment exposures, diagnostic approaches, and outcome measures across the African evidence base. Variability in access to healthcare services, timing of assessment, HIV/TB co-infection, ototoxic medication exposure, and differences in audiological and vestibular assessment methods may also have contributed to the wide range of reported prevalence estimates observed between studies.

Importantly, however, while the methodological heterogeneity limits direct comparison between studies, it also reflects the diversity of African healthcare systems, disease profiles, clinical pathways, and rehabilitation contexts. Consequently, the observed variability should be interpreted as an inherent characteristic of the current evidence base rather than solely as a methodological limitation. The included studies represented highly diverse populations across multiple countries, healthcare systems, and levels of care, where hearing-functioning problems are likely influenced by differing infectious disease burdens, chronic disease profiles, treatment pathways, and rehabilitation access. Despite variation in prevalence estimates, recurring multidimensional hearing-function profiles were consistently observed across conditions, particularly the co-occurrence of hearing loss, tinnitus, and vestibular dysfunction. These findings reinforce the importance of broader rehabilitation-oriented approaches capable of capturing overlapping auditory and vestibular disability across diverse African contexts.

### 4.6. Limitations and Strengths

The findings of this study should be interpreted within the context of the available African evidence base rather than as representative population prevalence estimates for hearing-functioning problems across the continent. The principal limitation of this study is the substantial methodological and clinical heterogeneity across the included studies. Differences in study design, populations, underlying health conditions, outcome measures, diagnostic criteria, and assessment methods likely contributed to the variability in reported prevalence estimates, which should therefore be interpreted as exploratory rather than representative of population-level prevalence. The evidence base also remained concentrated within particular regions, urban settings, hospital- and primary-care contexts, and infectious disease populations, potentially limiting broader generalisability across diverse African settings. As this study mapped the available published evidence, these patterns should be interpreted as reflecting the current distribution of research rather than the true distribution of hearing-functioning problems or rehabilitation needs across Africa.

Furthermore, restricting the parent scoping review to English-language publications may have resulted in the omission of relevant evidence published in other languages commonly used across Africa. Nevertheless, English remains the predominant language of health science publication across Africa, with substantially fewer publications available in French and Portuguese, suggesting that although relevant non-English evidence may have been missed, the overall impact on the mapped evidence base is likely to be limited [59]. The broader Rehab4All application is currently expanding multilingual data extraction capacity, including French and Portuguese literature, to improve representation of the African evidence base in future analyses.

Despite this heterogeneity, the use of the Rehab4All database enabled broader profiling of hearing-functioning problems across multiple health conditions and levels of care, allowing exploration of multidimensional and interconnected functional patterns that may not be apparent within single-condition or single-symptom studies alone. The use of a comprehensive, systematically extracted database, standardised ICF-based classification of functioning problems, and multidimensional analytical approaches further strengthened the analysis and provided an exploratory rehabilitation-relevant overview of hearing-function profiles associated with major health conditions contributing to disability in Africa. Importantly, by synthesising functioning problems across multiple high-burden health conditions, this study moves beyond traditional disease-specific descriptions to provide a rehabilitation-oriented understanding of hearing-functioning within the African evidence base.

### 4.7. Recommendations for Future Research

Future research should prioritise greater standardisation of hearing- and vestibular-function assessment approaches across African contexts to improve comparability between studies. There is also a need for increased research representation from under-represented regions, rural and community-based settings, and non-communicable disease populations. Longitudinal and multidisciplinary studies exploring the co-occurrence and progression of hearing-functioning problems across different health conditions may further strengthen understanding of rehabilitation needs and inform integrated hearing and rehabilitation service planning within African healthcare systems.

## 5. Conclusions

This scoping review mapped hearing-functioning problems among adults living with conditions contributing substantially to YLD across Africa using the Rehab4All database. The findings demonstrate that hearing-functioning problems within the African evidence base are multidimensional, interconnected, and contextually variable, extending beyond impairment-level hearing loss alone. Across several conditions, hearing loss frequently co-occurred with tinnitus, vestibular dysfunction, auditory-processing difficulties, and phonophobia, highlighting the broader functional and sensory dimensions of hearing-related disability. The findings further revealed important regional, contextual, and health-system disparities within the current evidence base, including the concentration of research within Southern Africa, urban settings, and infectious disease populations. Collectively, these findings highlight the need for broader rehabilitation-oriented approaches to hearing-function assessment and management within African contexts, while also underscoring the importance of expanding research beyond traditional impairment-focused models to better capture the complexity of hearing-related functioning across populations and settings.

## Figures and Tables

**Table 1:** Demographic details of included studies.

Study Design	No. of Articles (*n* = 97)
**Case–control**	5 (5%)
Case study/series	22 (23%)
Cohort	23 (24%)
Cross-Sectional	33 (34%)
Quasi-Experimental	3 (3%)
RCT	3 (3%)
Review	7 (7%)
Survey	1 (1%)
**Level of Care**	**No. of Articles**
Community level	8 (8%)
Hospital level	34 (35%)
Not reported	10 (10%)
Primary care clinic	29 (30%)
Rehabilitation facility	2 (2%)
Specialised hospital	14 (14%)
**Setting**	**No. of Articles**
Not reported	42 (43%)
Rural	5 (5%)
Rural + Urban	1 (1%)
Semi-Rural	1 (1%)
Urban	48 (50%)

**Table 2:** Number of articles covering hearing-functioning problems in top YLD-contributing conditions in Africa.

Condition	Disease Category	No. of Articles (*n*)
Cancer (other, specified)	Cancer (*n* = 2)	1
Cancer with hearing loss		1
DM (Diabetes Mellitus) with amputation	DM (*n* = 7)	1
DM, Type I		1
DM, Type II		2
DM, Type unspecified		3
Headache, Cervicogenic	Headaches (*n* = 7)	1
Headache, Migraine		5
Headache, unspecified		1
Hearing loss	Hearing Loss Cohorts (*n* = 20)	18
Hearing loss due to drug toxicity		1
Hearing loss with Genetic disorders		1
HIV/AIDS only	HIV/AIDS (*n* = 31)	29
HIV/AIDS with Fracture(s)		1
HIV/AIDS with Karposi’s sarcoma		1
HIV/AIDS with TB	HIV/AIDS + TB (*n* = 2)	2
TB, Multidrug-Resistant (MDR)	TB (*n* = 16)	15
TB, unspecified		1
Intracerebral haemorrhage	Stroke (*n* = 7)	1
Ischemic stroke		2
Stroke, other specified		1
Stroke, unspecified		3
Major depressive disorder	Mental Health (*n* = 1)	1
Neck pain	Musculoskeletal (*n* = 2)	2
Visually Impaired	Vision (*n* = 1)	1
War-affected	Conflict and terrorism (*n* = 1)	1

**Table 3:** Average prevalence (%) of hearing-functioning problems in conditions contributing to highest YLD in Africa.

Functioning Problem	Disease Category	TB	HIV/AIDS **	Hearing Loss Cohorts	Headaches	DM	Stroke	Cancer	Vision Impairment	Musculo-Skeletal	Depressive Disorders
**Vestibular dysfunction**	Av. Prevalence %	12.79	19.78	37.82	46.91	25.33	6.13	-	-	37.21	-
	Total Sample	191	5064	454	441	391	635	-	-	148	-
	No. of articles	3	20	7	4	4	3 (4 entries) *	-	-	2	-
**Tinnitus**	Av. Prevalence %	31.06	21.62	73.29	13.79	28.13	-	30.9	40	-	-
	Total Sample	360	985	789	58	331	-	16	5	-	-
	No. of articles	4	5	11	1	3	-	1	1	-	-
**Hearing Loss**	Av. Prevalence %	35.63	21.69	-	-	37.6	29.47	85.5	-	-	9.09
	Total Sample	3644	2202	-	-	441	343	47	-	-	33
	No. of articles	17	14	-	-	4	2	1	-	-	1
**Balance-related Problems**	Av. Prevalence %	-	27	-	-	21.84	-	-	-	-	-
	Total Sample	-	100	-	-	87	-	-	-	-	-
	No. of articles	-	2	-	-	1	-	-	-	-	-
**Other Auditory Symptoms**	Av. Prevalence %	-	-	88.41	-	-	-	-	-	-	-
	Total Sample	-	-	138	-	-	-	-	-	-	-
	No. of articles	-	-	1 (2 entries) *	-	-	-	-	-	-	-
**Auditory Processing**	Av. Prevalence %	-	-	90	-	-	-	-	-	-	-
	Total Sample	-	-	64	-	-	-	-	-	-	-
	No. of articles	-	-	2	-	-	-	-	-	-	-
**Phonophobia**	Av. Prevalence %	-	-	-	74.3	-	-	-	-	-	-
	Total Sample	-	-	-	223	-	-	-	-	-	-
	No. of articles	-	-	-	2	-	-	-	-	-	-

* Entries refer to if the article had more than one eligible population (e.g., ischemic and haemorrhagic stroke). ** HIV/AIDS column includes studies of HIV/AIDS only and HIV/AIDS-TB co-infection.

## Data Availability

The Rehab4All web-based application (https://ttlx.me/rehab4all; accessed on 21 July 2026) and its underlying data are the intellectual property of Stellenbosch University and remain under active development; therefore, they are not publicly available. Access may be considered upon reasonable request to the corresponding author (GD; gouwa@sun.ac.za) and subject to institutional approval under Stellenbosch University’s data governance and intellectual property policies. Requests will be considered, reviewed and actioned within 6 weeks of receipt. A complete list of publicly available studies included in the analysis, with DOIs, URLs or PubMed identifiers (PMIDs), is provided as Table A1 in Appendix A.

## Abbreviations

The following abbreviations are used in this manuscript:

DM	Diabetes Mellitus
GBD	Global Burden of Disease
HIV/AIDS	Human Immunodeficiency Virus/Acquired Immunodeficiency Syndrome
ICF	International Classification of Functioning, Disability and Health
TB	Tuberculosis
YLD	Years lived with disability
WHO	World Health Organisation

## Appendix A

Appendix A includes additional graphs and visual demonstrations of our findings for the reader’s enrichment.

**Table A1:** A complete list of publicly available studies included in the analysis, with DOIs, URLs or PubMed identifiers (PMIDs).

Article Name	URL	Reference
A comprehensive analysis of stroke admissions at a rural Nigerian tertiary health facility: Insights from a single-center study	https://doi.org/10.25259/JNRP_76_2023	[68]
A longitudinal community-based ototoxicity monitoring programme and treatment effects for drug-resistant tuberculosis treatment, Western Cape	https://doi.org/10.4102/sajcd.v69i1.886	[31]
A rare case of primary inverted papilloma of the middle ear	https://doi.org/10.11604/pamj.2019.33.49.18065	[69]
A rare tumour in the cerebellopontine angle: Endolymphatic sac tumour	https://doi.org/10.11604/pamj.2018.31.127.3962	[70]
Access to acute stroke care: A retrospective descriptive analysis of stroke patients’ journey to a district hospital	https://doi.org/10.1016/j.afjem.2022.07.010	[71]
Adverse effects profile of multidrug-resistant tuberculosis treatment in a South African outpatient clinic	https://doi.org/10.1080/20786204.2012.10874288	[32]
Adverse Events During Treatment of Drug-Resistant Tuberculosis: A Comparison Between Patients With or Without Human Immunodeficiency Virus Co-infection	https://doi.org/10.1007/s40264-013-0091-1	[33]
Amikacin Concentrations Predictive of Ototoxicity in Multidrug-Resistant Tuberculosis Patients	https://doi.org/10.1128/AAC.01050-15	[34]
Aminoglycoside-induced Hearing Loss Among Patients Being Treated for Drug-resistant Tuberculosis in South Africa: A Prediction Model	https://doi.org/10.1093/cid/ciz289	[45]
Aminoglycoside-induced hearing loss in HIV-positive and HIV-negative multidrug-resistant tuberculosis patients	https://doi.org/10.7196/SAMJ.4964	[35]
An Analysis of Risk Factors for Hearing Function in Adults Living with Human Immunodeficiency Virus in Gauteng, South Africa	https://doi.org/10.1007/s12070-023-04375-z	[60]
An assessment of adverse drug reactions among HIV positive patients receiving antiretroviral treatment in South Africa	https://doi.org/10.1186/s12981-015-0044-0	[13]
An unusual intracranial metallic foreign bodies and panhypopituitarism	https://doi.org/10.11604/pamj.2014.19.33.3862	[72]
Assessment of cochlear and auditory pathways in patients with migraine	https://doi.org/10.1016/j.amjoto.2011.10.008	[73]
Audiological profile of patients with type 2 diabetes mellitus	https://doi.org/10.4102/sajcd.v71i1.1035	[74]
Auditory and otological manifestations in adults with HIV/AIDS	https://doi.org/10.3109/14992027.2012.721935	[14]
Auditory Impairments in HIV-Infected Individuals in Tanzania	https://doi.org/10.1097/01.aud.0000439101.07257.ed	[61]
An analysis of auditory manifestations in a group of adults with AIDS prior to antiretroviral therapy	https://journals.athmsi.org/index.php/AJID/article/view/1167	[15]
Case Report: Kanamycin Ototoxicity and MDR-TB Treatment Regimen	https://doi.org/10.2147/IMCRJ.S336259	[75]
Case series of six patients diagnosed and managed for idiopathic intracranial hypertension at a tertiary institution eye centre	https://doi.org/10.4314/gmj.v53i1.12	[76]
Case study: New onset of neuropsychiatric symptoms following switching to a dolutegravir regimen	https://doi.org/10.4102/sajpsychiatry.v28i0.1782	[77]
Cerebellar stroke complicating coronary catheterization: A case report	https://doi.org/10.11604/pamj.2021.40.172.32031	[78]
Challenge to treat pre-extensively drug-resistant tuberculosis in a low-income country: A report of 12 cases	https://doi.org/10.1016/j.jctube.2020.100192	[36]
Chronic neck pain and vertigo: Is a true balance disorder present?	https://doi.org/10.1016/j.rehab.2009.07.033	[79]
Clinical and audiometric features of presbycusis in Nigerians	https://doi.org/10.4314/ahs.v13i4.4	[80]
Cochleo-vestibular clinical findings among drug resistant Tuberculosis Patients on therapy-a pilot study	https://doi.org/10.1186/1755-7682-5-3	[37]
Cognitive Disorders In Acquired Sensorineural Hearing Loss, At The Ent Department of The “Village Bondeko” Center, In Kinshasa	https://doi.org/10.2147/NDT.S478277	[81]
Common impairments and functional limitations of HIV sequelae that require physiotherapy rehabilitation in the medical wards at Queen Elizabeth Central Hospital, Malawi: A cross sectional study	https://doi.org/10.4314/mmj.v31i3.2	[16]
Comorbidities and Treatment Outcomes in Patients Diagnosed with Drug-Resistant Tuberculosis in Rural Eastern Cape Province, South Africa	https://doi.org/10.3390/diseases12110296	[38]
Comparing amikacin and kanamycin-induced hearing loss in multidrug-resistant tuberculosis treatment under programmatic conditions in a Namibian retrospective cohort	https://doi.org/10.1186/s40360-015-0036-7	[39]
Counselling and amplification with and without fractal music (Zen tones) for management of patients suffering from hearing loss and tinnitus	https://doi.org/10.1080/21695717.2017.1421812	[82]
Depressive symptoms among survivors of Ebola virus disease in Conakry (Guinea): Preliminary results of the PostEboGui cohort	https://doi.org/10.1186/s12888-017-1280-8	[83]
Discriminant Analysis of the Prognostic Factors for Hearing Outcomes in Patients with Idiopathic Sudden Sensorineural Hearing Loss	https://doi.org/10.5152/iao.2023.22893	[84]
Disseminated histoplasmosis diagnosed on a blood smear in a Nigerian patient with non-Hodgkin’s lymphoma	https://doi.org/10.1093/omcr/omae116	[85]
Do challenges still exist amongst HIV/AIDS patients in managing their condition? A cross-sectional study of 297 participants in the Ethekwini Metro of KwaZulu-Natal, South Africa	https://doi.org/10.2989/16085906.2019.1648305	[17]
Dolutegravir in real life: Self-reported mental and physical health outcomes after transitioning from efavirenz- to dolutegravir-based antiretroviral therapy in a prospective cohort study in Lesotho	https://doi.org/10.1111/hiv.13352	[18]
Effect of a tailored multidimensional intervention on the care burden among family caregivers of stroke survivors: A randomised controlled trial	https://doi.org/10.1136/bmjopen-2021-049741	[86]
Effect of HIV Infection and Highly Active Antiretroviral Therapy on Hearing Function	https://doi.org/10.1001/jamaoto.2015.125	[62]
Effect of Neural Mobilization on Nerve-Related Neck and Arm Pain: A Randomized Controlled Trial	https://doi.org/10.3138/ptc-2018-0056	[87]
Efficacy, safety, and tolerability of dolutegravir-based ART regimen in Durban, South Africa: A cohort study	https://doi.org/10.1186/s12879-024-09202-6	[19]
Electrophysiological differences in sensorineural hearing loss patients with and without problem-tinnitus	https://doi.org/10.7123/01.EJO.0000411078.05971.d1	[88]
Experience in endoscopic stapedotomy technique and its audiological outcome: A case series	https://doi.org/10.1186/s43163-021-00141-6	[89]
Exploring quality of life post sudden onset hearing loss: A convergent parallel approach	https://doi.org/10.4102/sajcd.v71i1.990	[90]
Eye movements and imaging in vestibular migraine	https://doi.org/10.1016/j.otorri.2018.10.001	[91]
Facial nerve function and general outcome of patients operated for cerebellopontine angle tumors in Addis Ababa, Ethiopia, a retrospective study	https://doi.org/10.1016/j.inat.2022.101612	[92]
Factors associated with physical function capacity in an urban cohort of people living with the human immunodeficiency virus in South Africa	https://doi.org/10.4102/sajp.v75i1.1323	[20]
Fall History and Associated Factors Among Adults Living With HIV-1 in the Cape Winelands, South Africa: An Exploratory Investigation	https://doi.org/10.1093/ofid/ofz401	[93]
Falls and associated risk factors in a sample of old age population in Egyptian community	https://doi.org/10.3389/fpubh.2023.1068314	[94]
Headaches in Consultation in the Workplace in Ouagadougou (Burkina Faso): Impact on the Quality of Life and the Patient’s Professional Performance	https://www.ajol.info/index.php/ajns/article/view/176386	[95]
Health Related Quality of Life and Sense of Coherence in Sudanese Diabetic Subjects with Lower Limb Amputation	https://doi.org/10.1620/tjem.217.45	[96]
Hearing impairment among chronic kidney disease patients on haemodialysis at a tertiary hospital in Ghana	https://doi.org/10.4314/gmj.v53i3.3	[97]
High prevalence of disabling hearing loss in young to middle-aged adults with diabetes	https://doi.org/10.1007/s13410-018-0655-9	[98]
HIV/AIDS-associated Kaposi’s sarcoma of the gastrointestinal tract: A pictorial spectrum	https://doi.org/10.7196/samj.2016.v106i10.11277	[99]
HIV-associated neurocognitive disorders: Antiretroviral regimen, central nervous system penetration effectiveness, and cognitive outcomes	https://doi.org/10.7196/samj.6677	[100]
HIV-related symptoms and management in HIV and antiretroviral therapy patients in KwaZulu-Natal, South Africa: A longitudinal study	https://doi.org/10.1080/17290376.2013.870119	[21]
Impact of drug-resistant tuberculosis treatment on hearing function in South African adults: Bedaquiline versus kanamycin	https://doi.org/10.4102/sajcd.v68i1.784	[40]
Investigation of physical and functional impairments experienced by people with active tuberculosis infection: A feasibility pilot study	https://doi.org/10.4102/ajod.v8i0.515	[41]
Investigation of the Interaction between Hearing Function and Comorbidities in Adults Living with Human Immunodeficiency Virus	https://doi.org/10.3390/ijerph182212177	[63]
Isolated Myxoma of the External Auditory Canal: A Case Report and Literature Review	https://doi.org/10.1177/0145561320927913	[101]
Isolated Sudden Bilateral Neurosensory Hearing Loss as a Presentation of Lyme Neuroborreliosis: A Case Study	https://doi.org/10.7874/jao.2023.00129	[102]
Maternally inherited diabetes and deafness with a variable presentation across three generations within a pedigree, South Africa	https://doi.org/10.4102/ajlm.v13i1.2384	[103]
Mental and physical health in Rwanda 14 years after the genocide	https://doi.org/10.1007/s00127-012-0494-9	[104]
Migraine among Egyptian medical students: Prevalence, disability and psychological distress-cross sectional study	https://doi.org/10.1186/s41983-023-00665-z	[105]
Migraine prevalence, clinical characteristics, and health care-seeking practice in a sample of medical students in Egypt	https://doi.org/10.1186/s41983-021-00282-8	[106]
Neuropsychiatric manifestations among HIV-1 infected African patients receiving efavirenz-based cART with or without tuberculosis treatment containing rifampicin	https://doi.org/10.1007/s00228-018-2499-0	[22]
Otolaryngological, head and neck manifestations in HIV-infected patients seen at Steve Biko Academic Hospital in Pretoria, South Africa	https://doi.org/10.7196/samj.6786	[23]
Otosyphilis: A rare cause of acute bilateral sensorineural hearing loss in a HIV-negative patient	https://doi.org/10.4102/sajr.v26i1.2351	[107]
Pathological vestibular symptoms presenting in a group of adults with HIV/AIDS in Johannesburg, South Africa	https://doi.org/10.4102/sajid.v32i2.53	[24]
Patient-Reported Adverse Effects Associated with Combination Antiretroviral Therapy and Coadministered Enzyme-Inducing Antiepileptic Drugs	https://doi.org/10.4269/ajtmh.16-0107	[25]
Peripheral neuropathy in HIV patients on antiretroviral therapy: Does it impact function?	https://doi.org/10.1016/j.jns.2019.116451	[26]
Pontobulbar palsy and sensorineural deafness (Brown-Vialetto-van Laere syndrome): The first case from Libya	https://doi.org/10.3109/17482960903440775	[108]
Predictive factors for recovery in idiopathic sudden sensory neural hearing loss	https://doi.org/10.1186/s43163-022-00348-1	[109]
Predictors of patient length of stay post stroke rehabilitation	https://doi.org/10.4314/ahs.v23i2.63	[110]
Pregnancies complicated by multidrug-resistant tuberculosis and HIV co-infection in Durban, South Africa	https://www.ingentaconnect.com/content/iuatld/ijtld/2007/00000011/00000006/art00020, accessed on 21 July 2026	[64]
Prevalence of hearing loss and tinnitus in a group of adults with Human Immunodeficiency Virus	https://doi.org/10.4102/sajcd.v67i1.631	[66]
Prevalence of ototoxicity in University of Benin Teaching Hospital, Benin city: A 5-year review	https://doi.org/10.4103/1119-3077.104527	[111]
Prevalence of Peripheral Vestibular Impairment in Adults with Human Immunodeficiency Virus	https://doi.org/10.7874/jao.2020.00164	[27]
Prevalence of Pre-Existing Hearing Loss Among Patients with Drug-Resistant Tuberculosis in South Africa	https://doi.org/10.1044/2020_AJA-19-00103	[46]
Primary Pleomorphic Adenoma of the Middle Ear and Mastoid	https://doi.org/10.1177/014556131809700908	[112]
Role of cervical vestibular-evoked myogenic potentials testing in vestibular migraine	https://doi.org/10.1016/j.ejenta.2015.04.001	[113]
Safety of antituberculosis agents used for multidrug-resistant tuberculosis among patients attending the Jamot Hospital of Yaounde, Cameroon	https://doi.org/10.4103/ijmy.ijmy_88_23	[42]
Stroke signs knowledge and factors associated with a delayed hospital arrival of patients with acute stroke in Kinshasa	https://doi.org/10.1016/j.heliyon.2024.e28311	[114]
Successful MDR-TB treatment regimens including Amikacin are associated with high rates of hearing loss	https://doi.org/10.1186/1471-2334-14-542	[43]
Suicidal overdose of dolutegravir: A case report	https://doi.org/10.4102/sajhivmed.v19i1.799	[115]
Surgical management of vestibular schwannoma: Attempted preservation of hearing and facial function	https://doi.org/10.1017/S0022215113000546	[116]
The effect of direct application of dexamethasone on the round window membrane in patients with sudden sensorineural hearing loss	https://doi.org/10.4103/ejo.ejo_49_17	[117]
The spectrum of hearing abnormalities in patients living with diabetes mellitus	https://doi.org/10.7196/SAMJ.2021.v111i10.15863	[118]
Treatment Outcomes and Adverse Events from a Standardized Multidrug-Resistant Tuberculosis Regimen in a Rural Setting in Angola	https://doi.org/10.4269/ajtmh.19-0175	[65]
Treatment outcomes of multi drug resistant and rifampicin resistant Tuberculosis in Zimbabwe: A cohort analysis of patients initiated on treatment during 2010 to 2015	https://doi.org/10.1371/journal.pone.0230848	[44]
Tympanometric Findings among Adult HIV Patients Undergoing a Short-Term Treatment with High Active Antiretroviral Therapy (HAART) in Port Harcourt	https://doi.org/10.4103/njcp.njcp_427_22	[28]
Auditory, video head impulse test and vestibular evoked myogenic potentials findings in adults with human immunodeficiency virus	https://doi.org/10.1016/j.anl.2019.11.006	[29]
Vestibular function assessment in cochlear implant patients	https://doi.org/10.4103/ejo.ejo_55_18	[119]
Vestibular Function in a Group of Adults with HIV/AIDS on HAART	https://doi.org/10.21010/ajid.v12i1.2	[120]
Vestibular involvement in adults with HIV/AIDS	https://doi.org/10.1016/j.anl.2013.08.003	[30]

## References

[1] World Health Organization Status Report on Ear and Hearing Care in the WHO African Region. https://www.afro.who.int/publications/status-report-ear-and-hearing-care-who-african-region.

[2] Ahmed A., Tsiga-Ahmed F., Bello-Muhammad N., Ajiya A., Gudaji M., Stefler D. (2025). Association of hearing loss with cognitive function and mental health in Africa: A systematic review. BMC Public Health.

[3] WHO Towards a Common Language for Functioning, Disability and Health: ICF. https://www.who.int/publications/m/item/icf-beginner-s-guide-towards-a-common-language-for-functioning-disability-and-health.

[4] Lasisi A.O., Gureje O. (2013). Disability and Quality of Life among Elderly Persons with Self-Reported Hearing Impairment: Report from the Ibadan Study of Aging. Int. J. Otolaryngol. Head Neck Surg..

[5] Govender S.M., de Jongh M. (2021). Identifying hearing impairment and the associated impact on the quality of life among the elderly residing in retirement homes in Pretoria, South Africa. S. Afr. J. Commun. Disord..

[6] Mnguni L.K., Mahomed O. (2026). Determinants of quality of life amongst hearing impaired patients in a rural district of South Africa: A cross-sectional study. Discov. Soc. Sci. Health.

[7] Baratedi W.M., Tshiamo W.B., Mokotedi M.T., Khutjwe J.V., Mamalelala T.T., Sewane E.B.P. (2022). Experiences of accessing healthcare services by people with hearing loss/impairment (deaf) in sub-Saharan Africa: An integrative review. J. Nurs. Scholarsh..

[8] Charumbira M.Y., Berner K., Blaauw D., Louw Q.A. (2022). Development of an innovative strategy to determine functioning attributed to health conditions in low-resource settings. Digit. Health.

[9] Charumbira M.Y., Berner K., Louw Q.A. (2022). Functioning Problems Associated with Health Conditions with Greatest Disease Burden in South Africa: A Scoping Review. Int. J. Environ. Res. Public Health.

[10] Arksey H., O’Malley L. (2005). Scoping studies: Towards a methodological framework. Int. J. Soc. Res. Methodol..

[11] Peters M.D.J., Marnie C., Tricco A.C., Pollock D., Munn Z., Alexander L., McInerney P., Godfrey C.M., Khalil H. (2020). Updated methodological guidance for the conduct of scoping reviews. JBI Evid. Synth..

[12] Charumbira M., Maart R., Bedada D., Joseph C., Louw Q. (2026). A scoping review and meta-analysis of functioning problems across 41 African countries. Nat. Health.

[13] Masenyetse L.J., Manda S.O., Mwambi H.G. (2015). An assessment of adverse drug reactions among HIV positive patients receiving antiretroviral treatment in South Africa. AIDS Res. Ther..

[14] van der Westhuizen Y., Swanepoel D.W., Heinze B., Hofmeyr L.M. (2013). Auditory and otological manifestations in adults with HIV/AIDS. Int. J. Audiol..

[15] Khoza-Shangase K. (2011). An analysis of auditory manifestations in a group of adults with AIDS prior to antiretroviral therapy. Afr. J. Infect. Dis..

[16] Banda G.T. (2019). Common impairments and functional limitations of HIV sequelae that require physiotherapy rehabilitation in the medical wards at Queen Elizabeth Central Hospital, Malawi: A cross sectional study. Malawi Med. J..

[17] Naidoo P., Premdutt R. (2019). Do challenges still exist amongst HIV/AIDS patients in managing their condition? A cross-sectional study of 297 participants in the Ethekwini Metro of KwaZulu-Natal, South Africa. Afr. J. AIDS Res..

[18] Brown J.A., Nsakala B.L., Mokhele K., Rakuoane I., Muhairwe J., Glass T.R., Amstutz A., Tschumi N., Belus J.M., Klimkait T. (2022). Dolutegravir in real life: Self-reported mental and physical health outcomes after transitioning from efavirenz- to dolutegravir-based antiretroviral therapy in a prospective cohort study in Lesotho. HIV Med..

[19] Hurbans N., Naidoo P. (2024). Efficacy, safety, and tolerability of dolutegravir-based ART regimen in Durban, South Africa: A cohort study. BMC Infect. Dis..

[20] Roos R., Myezwa H., Van Aswegen H. (2019). Factors associated with physical function capacity in an urban cohort of people living with the human immunodeficiency virus in South Africa. S. Afr. J. Physiother..

[21] Peltzer K. (2013). HIV-related symptoms and management in HIV and antiretroviral therapy patients in KwaZulu-Natal, South Africa: A longitudinal study. SAHARA-J J. Soc. Asp. HIV/AIDS.

[22] Mugusi S., Ngaimisi E., Janabi M., Mugusi F., Minzi O., Aris E., Bakari M., Bertilsson L., Burhenne J., Sandstrom E. (2018). Neuropsychiatric manifestations among HIV-1 infected African patients receiving efavirenz-based cART with or without tuberculosis treatment containing rifampicin. Eur. J. Clin. Pharmacol..

[23] Tshifularo M., Govender L., Monama G. (2013). Otolaryngological, head and neck manifestations in HIV-infected patients seen at Steve Biko Academic Hospital in Pretoria, South Africa. S. Afr. Med. J..

[24] Khoza-Shangase K., Van Rie K.J. (2017). Pathological vestibular symptoms presenting in a group of adults with HIV/AIDS in Johannesburg, South Africa. S. Afr. J. Infect. Dis..

[25] Elafros M.A., Birbeck G.L., Gardiner J.C., Siddiqi O.K., Sikazwe I., Paneth N., Bositis C.M., Okulicz J.F. (2017). Patient-Reported Adverse Effects Associated with Combination Antiretroviral Therapy and Coadministered Enzyme-Inducing Antiepileptic Drugs. Am. Soc. Trop. Med. Hyg..

[26] Dudley M.T., Borkum M., Basera W., Wearne N., Heckmann J.M. (2019). Peripheral neuropathy in HIV patients on antiretroviral therapy: Does it impact function?. J. Neurol. Sci..

[27] Millar A., Joubert K., Naude A. (2021). Prevalence of Peripheral Vestibular Impairment in Adults with Human Immunodeficiency Virus. J. Audiol. Otol..

[28] Ejikeme U., da Lilly-Tariah O., Onotai L., Chinenye S. (2023). Tympanometric Findings among Adult HIV Patients Undergoing a Short-Term Treatment with High Active Antiretroviral Therapy (HAART) in Port Harcourt. Niger. J. Clin. Pract..

[29] Mahomed W., Heinze B.M., Vinck B.H., Stoltz A. (2019). Auditory, video head impulse test and vestibular evoked myogenic potentials findings in adults with human immunodeficiency virus. Auris Nasus Larynx.

[30] Heinze B.M., Vinck B.M., Hofmeyr L.M., Swanepoel D.W. (2014). Vestibular involvement in adults with HIV/AIDS. Auris Nasus Larynx.

[31] Stevenson L.J., Biagio-de Jager L., Graham M.A., Swanepoel D.W. (2022). A longitudinal community-based ototoxicity monitoring programme and treatment effects for drug-resistant tuberculosis treatment, Western Cape. S. Afr. J. Commun. Disord..

[32] Jacobs T., Ross A. (2012). Adverse effects profile of multidrug-resistant tuberculosis treatment in a South African outpatient clinic. S. Afr. Fam. Pract..

[33] Sagwa E., Ruswa N., Musasa J.P., Mantel-Teeuwisse A.K. (2013). Adverse Events During Treatment of Drug-Resistant Tuberculosis: A Comparison Between Patients With or Without Human Immunodeficiency Virus Co-infection. Drug Saf..

[34] Modongo C., Pasipanodya J.G., Zetola N.M., Williams S.M., Sirugo G., Gumbo T. (2015). Amikacin Concentrations Predictive of Ototoxicity in Multidrug-Resistant Tuberculosis Patients. Antimicrob. Agents Chemother..

[35] Harris T., Bardien S., Schaaf H.S., Petersen L., De Jong G., Fagan J.J. (2012). Aminoglycoside-induced hearing loss in HIV-positive and HIV-negative multidrug-resistant tuberculosis patients. S. Afr. Med. J..

[36] Murhula Kashongwe I., Mawete F., Anshambi N., Maingowa N., Aloni M., L’OSenga L.L., Kaswa M., Kashongwe Z.M. (2020). Challenge to treat pre-extensively drug-resistant tuberculosis in a low-income country: A report of 12 cases. J. Clin. Tuberc. Other Mycobact. Dis..

[37] Ramma L., Ibekwe T.S. (2012). Cochleo-vestibular clinical findings among drug resistant Tuberculosis Patients on therapy-a pilot study. Int. Arch. Med..

[38] Hosu M.C., Faye L.M., Apalata T. (2024). Comorbidities and Treatment Outcomes in Patients Diagnosed with Drug-Resistant Tuberculosis in Rural Eastern Cape Province, South Africa. Diseases.

[39] Sagwa E.L., Ruswa N., Mavhunga F., Rennie T., Leufkens H.G.M., Mantel-Teeuwisse A.K. (2015). Comparing amikacin and kanamycin-induced hearing loss in multidrug-resistant tuberculosis treatment under programmatic conditions in a Namibian retrospective cohort. BMC Pharmacol. Toxicol..

[40] Khoza-Shangase K., Prodromos M. (2021). Impact of drug-resistant tuberculosis treatment on hearing function in South African adults: Bedaquiline versus kanamycin. S. Afr. J. Commun. Disord..

[41] Van Aswegen H., Roos R., McCree M., Quinn S., Mer M. (2019). Investigation of physical and functional impairments experienced by people with active tuberculosis infection: A feasibility pilot study. Afr. J. Disabil..

[42] Mpoh M., Deli V., Daniel T., Salvo F. (2023). Safety of antituberculosis agents used for multidrug-resistant tuberculosis among patients attending the Jamot Hospital of Yaounde, Cameroon. Int. J. Mycobacteriol..

[43] Modongo C., Sobota R.S., Kesenogile B., Ncube R., Sirugo G., Williams S.M., Zetola N.M. (2014). Successful MDR-TB treatment regimens including Amikacin are associated with high rates of hearing loss. BMC Infect. Dis..

[44] Matambo R., Takarinda K.C., Thekkur P., Sandy C., Mharakurwa S., Makoni T., Ncube R., Charambira K., Zishiri C., Ngwenya M. (2020). Treatment outcomes of multi drug resistant and rifampicin resistant Tuberculosis in Zimbabwe: A cohort analysis of patients initiated on treatment during 2010 to 2015. PLoS ONE.

[45] Hong H., Dowdy D.W., E Dooley K., Francis H.W., Budhathoki C., Han H.-R., Farley J.E. (2019). Aminoglycoside-induced Hearing Loss Among Patients Being Treated for Drug-resistant Tuberculosis in South Africa: A Prediction Model. Clin. Infect. Dis..

[46] Hong H., Dowdy D.W., Dooley K.E., Francis H.W., Budhathoki C., Han H.-R., Farley J.E. (2020). Prevalence of Pre-Existing Hearing Loss Among Patients with Drug-Resistant Tuberculosis in South Africa. Am. J. Audiol..

[47] Ihler F., Brzoska T., Altindal R., Dziemba O., Völzke H., Busch C.-J., Ittermann T. (2024). Prevalence and risk factors of self-reported hearing loss, tinnitus, and dizziness in a population-based sample from rural northeastern Germany. Sci. Rep..

[48] Liu C., Wu X., Li J., Zhang J., Gao Y., Wang D., Wang Q. (2025). Comorbidity Patterns Between Hearing Loss and Symptomatic Dizziness in Middle-Aged and Older Adults. World J. Otorhinolaryngol. Head Neck Surg..

[49] Hong H., Budhathoki C., Farley J.E. (2018). Increased risk of aminoglycoside-induced hearing loss in MDR-TB patients with HIV coinfection. Int. J. Tuberc. Lung Dis..

[50] Hong H., Dowdy D.W., Dooley K.E., Francis H.W., Budhathoki C., Han H.-R., Farley J.E. (2020). Risk of hearing loss among multidrug-resistant tuberculosis patients according to cumulative aminoglycoside dose. Int. J. Tuberc. Lung Dis..

[51] Heinze B., Swanepoel D.W., Hofmeyr L.M. (2011). Systematic review of vestibular disorders related to human immunodeficiency virus and acquired immunodeficiency syndrome. J. Laryngol. Otol..

[52] Kumar P., Singh N.K., Apeksha K., Ghosh V., Kumar R.R., Kumar Muthaiah B. (2022). Auditory and Vestibular Functioning in Individuals with Type-2 Diabetes Mellitus: A Systematic Review. Int. Arch. Otorhinolaryngol..

[53] Cantillo-Martínez M., Lorente-Piera J., Manrique-Huarte R., Sánchez-Del-Río M., Pérez-Fernández N., Chico-Vila C., Moreno-Ajona D., Irimia P. (2025). Insights into Vestibular Migraine: Diagnostic Challenges, Differential Spectrum and Therapeutic Horizons. J. Clin. Med..

[54] Olusanya B.O. (2004). Classification of childhood hearing impairment: Implications for rehabilitation in developing countries. Disabil. Rehabil..

[55] Rogers C. (2021). Audiologists should not fail with falls: A call to commit to prevention of falls in older adults. S. Afr. J. Commun. Disord..

[56] Agrawal Y., Smith P.F., Rosenberg P.B. (2020). Vestibular impairment, cognitive decline and Alzheimer’s disease: Balancing the evidence. Aging Ment. Health.

[57] Li C.-M., Hoffman H.J., Ward B.K., Della Santina C.C., Cotch M.F., Chiu M.S., Losonczy K.G., Themann C.L., Launer L.J., Siggeirsdottir K. (2025). Impact of Balance and Dizziness Problems on Falls in Older Adults: The Longitudinal AGES-Reykjavik Study. Laryngoscope Investig. Otolaryngol..

[58] UNAIDS The Path That Ends AIDS: UNAIDS Global AIDS Update 2023. Geneva: Joint United Nations Programme on HIV/AIDS. https://www.unaids.org/en/resources/documents/2023/global-aids-update-2023.

[59] Yarmoshuk A.N., Mloka D., Fidèle Touré S., Sharma V., Wanji S., Chan H.T.H. (2021). Research into Language-Based Equity in African Health Science Research. https://cms.wellcome.org/sites/default/files/2021-06/language-based-equity-in-african-health-science-research.pdf.

[60] Sebothoma B. (2024). An Analysis of Risk Factors for Hearing Function in Adults Living with Human Immunodeficiency Virus in Gauteng, South Africa. Indian J. Otolaryngol. Head Neck Surg..

[61] Maro I.I., Moshi N., Clavier O.H., MacKenzie T.A., Kline-Schoder R.J., Wilbur J.C., Chambers R.D., Fellows A.M., Jastrzembski B.G., Mascari J.E. (2014). Auditory Impairments in HIV-Infected Individuals in Tanzania. Ear Hear..

[62] Fokouo J.V.F., Vokwely J.E.E., Noubiap J.J.N., Nouthe B.E., Zafack J., Ngom E.S.M., Dalil A.B., Nyeki A.-R.N., Bengono G., Njock R. (2015). Effect of HIV Infection and Highly Active Antiretroviral Therapy on Hearing Function. JAMA Otolaryngol.-Head Neck Surg..

[63] Sebothoma B., Khoza-Shangase K. (2021). Investigation of the Interaction between Hearing Function and Comorbidities in Adults Living with Human Immunodeficiency Virus. Int. J. Environ. Res. Public Health.

[64] Khan M., Pillay T., Moodley J., Ramjee A., Padayatchi N. (2007). Pregnancies complicated by multidrug-resistant tuberculosis and HIV co-infection in Durban, South Africa. Int. J. Tuberc. Lung Dis..

[65] Aznar M.L., Segura A.R., Moreno M.M., Espasa M., Sulleiro E., Bocanegra C., Gil Olivas E., Eugénio A.N., Zacarias A., Katimba D. (2019). Treatment Outcomes and Adverse Events from a Standardized Multidrug-Resistant Tuberculosis Regimen in a Rural Setting in Angola. Am. J. Trop. Med. Hyg..

[66] Millar A., Joubert K., Naude A. (2020). Prevalence of hearing loss and tinnitus in a group of adults with Human Immunodeficiency Virus. S. Afr. J. Commun. Disord..

[67] Spooner R., Ranasinghe S., Urasa S., Yoseph M., Koipapi S., Mukaetova-Ladinska E.B., Lewis T., Howlett W., Dekker M., Kisoli A. (2022). HIV-Associated Neurocognitive Disorders: The First Longitudinal Follow-Up of a cART-Treated Cohort of Older People in Sub-Saharan Africa. J. Acquir. Immune Defic. Syndr..

[68] Erameh C.O., Emorinken A., Akpasubi B.O. (2023). A comprehensive analysis of stroke admissions at a rural Nigerian tertiary health facility: Insights from a single-center study. J. Neurosci. Rural Pract..

[69] Hasnaoui M., Masmoudi M., Ben Abdeljelil N., Ben Hmida N., Driss N. (2019). A rare case of primary inverted papilloma of the middle ear. Pan Afr. Med. J..

[70] Elktaibi A., Damiri A., Rharrassi I., Elochi M.R., Oukabli M., Akhaddar A., Boucetta M., Al Bouzidi A. (2018). A rare tumour in the cerebellopontine angle: Endolymphatic sac tumour. Pan Afr. Med. J..

[71] Mark O’Meara R., Ganas U., Hendrikse C. (2022). Access to acute stroke care: A retrospective descriptive analysis of stroke patients’ journey to a district hospital. Afr. J. Emerg. Med..

[72] Lakouichmi M., Baïzri H., Mouhsine A., Boukhira A., Akhaddar A. (2014). An unusual intracranial metallic foreign bodies and panhypopituitarism. Pan Afr. Med. J..

[73] Hamed S.A., Youssef A.H., Elattar A.M. (2012). Assessment of cochlear and auditory pathways in patients with migraine. Am. J. Otolaryngol..

[74] Nkosi S.T., Peter V.Z., Paken J. (2024). Audiological profile of patients with type 2 diabetes mellitus. S. Afr. J. Commun. Disord..

[75] Mantefardo B., Sisay G. (2021). Case Report: Kanamycin Ototoxicity and MDR-TB Treatment Regimen. Int. Med. Case Rep. J..

[76] Tagoe N.N., Beyuo V.M., Amissah-Arthur K.N. (2019). Case series of six patients diagnosed and managed for idiopathic intracranial hypertension at a tertiary institution eye centre. Ghana Med. J..

[77] Badat A., Lowton K. (2022). Case study: New onset of neuropsychiatric symptoms following switching to a dolutegravir regimen. S. Afr. J. Psychiatry.

[78] Chraibi H., El Yousfi Z., Mouine N., Lakhal Z., Benyass A. (2021). Cerebellar stroke complicating coronary catheterization: A case report. Pan Afr. Med. J..

[79] Yahia A., Ghroubi S., Jribi S., Mâlla J., Baklouti S., Ghorbel A., Elleuch M. (2009). Chronic neck pain and vertigo: Is a true balance disorder present?. Ann. Phys. Rehabil. Med..

[80] Sogebi O., Olusoga-Peters O., Oluwapelumi O. (2014). Clinical and audiometric features of presbycusis in Nigerians. Afr. Health Sci..

[81] Masamba G., Gedikondele J.S., Longo-Mbenza B., Nkanga M.S.N., Nzanza R.M., Matonda-Ma-Nzuzi T., Ikanga J., Okwe A.N., Mabwaka G.L., Malengele H.M. (2025). Cognitive Disorders In Acquired Sensorineural Hearing Loss, At The Ent Department of The “Village Bondeko” Center, In Kinshasa. Neuropsychiatr. Dis. Treat..

[82] Shabana M.I., Dabbous A.O., Abdelmajeed M.A., Abdelkarim A.M.M. (2018). Counselling and amplification with and without fractal music (Zen tones) for management of patients suffering from hearing loss and tinnitus. Hear. Balance Commun..

[83] Keita M.M., Taverne B., Savané S.S., March L., Doukoure M., Sow M.S., Touré A., Etard J.F., Barry M., Delaporte E. (2017). Depressive symptoms among survivors of Ebola virus disease in Conakry (Guinea): Preliminary results of the PostEboGui cohort. BMC Psychiatry.

[84] Askar A.A., Ghonim M.R., Kamel Shabana Y. (2023). Discriminant Analysis of the Prognostic Factors for Hearing Outcomes in Patients with Idiopathic Sudden Sensorineural Hearing Loss. J. Int. Adv. Otol..

[85] Benjamin O.O., Riman O.E., Offiong A., Egbara W.O., Onukak A., Ogar A.N., Ekeng B.E. (2024). Disseminated histoplasmosis diagnosed on a blood smear in a Nigerian patient with non-Hodgkin’s lymphoma. Oxf. Med. Case Rep..

[86] Elsheikh M.A., Moriyama M., Rahman M., Kako M., El-Monshed A.H., Zoromba M., Zehry H., Khalil M.H., El-Gilany A.-H., Amr M. (2022). Effect of a tailored multidimensional intervention on the care burden among family caregivers of stroke survivors: A randomised controlled trial. BMJ Open.

[87] Basson C.A., Stewart A., Mudzi W., Musenge E. (2020). Effect of Neural Mobilization on Nerve-Related Neck and Arm Pain: A Randomized Controlled Trial. Physiother. Can..

[88] Said E.A. (2012). Electrophysiological differences in sensorineural hearing loss patients with and without problem-tinnitus. Egypt. J. Otolaryngol..

[89] Abdullah N.E., Nafie T.A., Mohammed A.F., Abdelmomin A.A., Yagi H.I., Ahmed A.M. (2021). Experience in endoscopic stapedotomy technique and its audiological outcome: A case series. Egypt. J. Otolaryngol..

[90] Ntlhakana L., Hamid S. (2024). Exploring quality of life post sudden onset hearing loss: A convergent parallel approach. S. Afr. J. Commun. Disord..

[91] ElSherif M., Reda M.I., Saadallah H., Mourad M. (2020). Eye movements and imaging in vestibular migraine. Acta Otorrinolaringol. Esp..

[92] Yesehak B., Tirsit A. (2022). Facial nerve function and general outcome of patients operated for cerebellopontine angle tumors in Addis Ababa, Ethiopia, a retrospective study. Interdiscip. Neurosurg..

[93] Berner K., Strijdom H., Essop M.F., Webster I., Morris L., Louw Q. (2019). Fall History and Associated Factors Among Adults Living With HIV-1 in the Cape Winelands, South Africa: An Exploratory Investigation. Open Forum Infect. Dis..

[94] El Sayed A.E.H.I., Said M.T., Mohsen O., Abozied A.M., Salama M. (2023). Falls and associated risk factors in a sample of old age population in Egyptian community. Front. Public Health.

[95] Bassole P.-R., Napon C., Kabore J., Ndiaye M.M. (2017). Headaches in Consultation in the Workplace in Ouagadougou (Burkina Faso): Impact on the Quality of Life and the Patient’s Professional Performance. Afr. J. Neurol. Sci..

[96] Abdelgadir M., Shebeika W., Eltom M., Berne C., Wikblad K. (2009). Health Related Quality of Life and Sense of Coherence in Sudanese Diabetic Subjects with Lower Limb Amputation. Tohoku J. Exp. Med..

[97] Boateng J.O., Boafo N., Osafo C., Anim-Sampong S. (2019). Hearing impairment among chronic kidney disease patients on haemodialysis at a tertiary hospital in Ghana. Ghana Med. J..

[98] Hlayisi V.-G., Petersen L., Ramma L. (2019). High prevalence of disabling hearing loss in young to middle-aged adults with diabetes. Int. J. Diabetes Dev. Ctries..

[99] Patel N., Naidoo P., Mosiane P., Jann-Kruger C. (2016). HIV/AIDS-associated Kaposi’s sarcoma of the gastrointestinal tract: A pictorial spectrum. S. Afr. Med. J..

[100] Cross H.M., Combrinck M.I., Joska J.A. (2013). HIV-associated neurocognitive disorders: Antiretroviral regimen, central nervous system penetration effectiveness, and cognitive outcomes. S. Afr. Med. J..

[101] Hasnaoui M., Masmoudi M., Belaid T., Mighri K. (2021). Isolated Myxoma of the External Auditory Canal: A Case Report and Literature Review. Ear Nose Throat J..

[102] Rochd S., Benhoummad O., Salhi S., Lakhdar Y., Rochdi Y., Raji A., Oualhadj H., El Kamouni Y., Zouhair S. (2024). Isolated Sudden Bilateral Neurosensory Hearing Loss as a Presentation of Lyme Neuroborreliosis: A Case Study. J. Audiol. Otol..

[103] Makgopa H., Kemp T., Meldau S., Honey E.M., Chale-Matsau B. (2024). Maternally inherited diabetes and deafness with a variable presentation across three generations within a pedigree, South Africa. Afr. J. Lab. Med..

[104] Munyandamutsa N., Mahoro Nkubamugisha P., Gex-Fabry M., Eytan A. (2012). Mental and physical health in Rwanda 14 years after the genocide. Soc. Psychiatry Psychiatr. Epidemiol..

[105] Ragab S., Zaitoun N., Elrafie A., El-Ansarey H., Srour A., Nabil N., Elshoura Y., Elshafei M., Elgamal S. (2023). Migraine among Egyptian medical students: Prevalence, disability and psychological distress-cross sectional study. Egypt. J. Neurol. Psychiatr. Neurosurg..

[106] Oraby M.I., Soliman R.H., Mahmoud M.A., Elfar E., Abd ElMonem N.A. (2021). Migraine prevalence, clinical characteristics, and health care-seeking practice in a sample of medical students in Egypt. Egypt. J. Neurol. Psychiatr. Neurosurg..

[107] Sothmann J., Adam S., van Tonder G., Davis R., van Rensburg L.J. (2022). Otosyphilis: A rare cause of acute bilateral sensorineural hearing loss in a HIV-negative patient. S. Afr. J. Radiol..

[108] Dakhil F.O., Bensreiti S.M., Zew M.H. (2010). Pontobulbar palsy and sensorineural deafness (Brown-Vialetto-van Laere syndrome): The first case from Libya. Amyotroph. Lateral Scler..

[109] Alhussaini M.A., Mohamed S.A., El-Razek M.A.A., Mohamed E.S., Gad M.O.A. (2022). Predictive factors for recovery in idiopathic sudden sensory neural hearing loss. Egypt. J. Otolaryngol..

[110] Bijl T., Mudzi W., Comley-White N. (2023). Predictors of patient length of stay post stroke rehabilitation. Afr. Health Sci..

[111] Obasikene G., Adobamen P., Okundia P., Ogusi F. (2012). Prevalence of ototoxicity in University of Benin Teaching Hospital, Benin city: A 5-year review. Niger. J. Clin. Pract..

[112] Bitew A., Sahle T., Redleaf M. (2018). Primary Pleomorphic Adenoma of the Middle Ear and Mastoid. Ear Nose Throat J..

[113] Mohamed E.S., Ahmed M.A.R., Said E.A.-F. (2015). Role of cervical vestibular-evoked myogenic potentials testing in vestibular migraine. Egypt. J. Ear Nose Throat Allied Sci..

[114] Kazadi Kabanda I., Ngonzo C.K., Bowamou C.-K.E., Nzambi J.-P.D., Ponte N.K., Madoda O.T., Natuhoyila A.N., M’BUyamba-Kabangu J.-R., Longo-Mbenza B., Bomba D.B. (2024). Stroke signs knowledge and factors associated with a delayed hospital arrival of patients with acute stroke in Kinshasa. Heliyon.

[115] Daimari R., Kwape L., Oyekunle A.A. (2018). Suicidal overdose of dolutegravir: A case report. S. Afr. J. HIV Med..

[116] Youssef T.F., Matter A., Ahmed M.R. (2013). Surgical management of vestibular schwannoma: Attempted preservation of hearing and facial function. J. Laryngol. Otol..

[117] Abdel Baki F.A., Omran A.A., Asal S.I., Hassan K.M. (2017). The effect of direct application of dexamethasone on the round window membrane in patients with sudden sensorineural hearing loss. Egypt. J. Otolaryngol..

[118] Pillay S., Naidoo K.H., Msimang K. (2021). The spectrum of hearing abnormalities in patients living with diabetes mellitus. S. Afr. Med. J..

[119] El-Karaksy A.A.R.M., Kouzo H.S., Attallah M.B., Talaat M.A. (2019). Vestibular function assessment in cochlear implant patients. Egypt. J. Otolaryngol..

[120] Khoza-Shangase K. (2018). Vestibular Function in a Group of Adults with HIV/AIDS on HAART. Afr. J. Infect. Dis..

